# Recent Advances in Brachytherapy Using Radioactive Nanoparticles: An Alternative to Seed-Based Brachytherapy

**DOI:** 10.3389/fonc.2021.766407

**Published:** 2021-11-24

**Authors:** Baljeet Seniwal, Velaphi C. Thipe, Sukhvir Singh, Telma C. F. Fonseca, Lucas Freitas de Freitas

**Affiliations:** ^1^ Centre de Recherche du Centre Hospitalier Universitaire de Québec-Université Laval (CR-CHU de Québec), Axe Médecine Régénératrice, Québec, QC, Canada; ^2^ Instituto de Pesquisas Energéticas e Nucleares, Comissão Nacional de Energia Nuclear (IPEN-CNEN), Cidade Universitária, São Paulo, Brazil; ^3^ Department of Radiology, Institute of Green Nanotechnology, School of Medicine, University of Missouri, Columbia, MO, United States; ^4^ Institute of Nuclear Medicine and Allied Sciences, Defence Research and Development Organisation, Delhi, India; ^5^ Departamento de Engenharia Nuclear—Universidade Federal de Minas Gerais, Belo Horizonte, Brazil

**Keywords:** interstitial brachytherapy, nanobrachytherapy, radioactive nanoparticles, intratumoral injection, solid tumors

## Abstract

Interstitial brachytherapy (BT) is generally used for the treatment of well-confined solid tumors. One example of this is in the treatment of prostate tumors by permanent placement of radioactive seeds within the prostate gland, where low doses of radiation are delivered for several months. However, successful implementation of this technique is hampered due to several posttreatment adverse effects or symptoms and operational and logistical complications associated with it. Recently, with the advancements in nanotechnology, radioactive nanoparticles (radio-NPs) functionalized with tumor-specific biomolecules, injected intratumorally, have been reported as an alternative to seed-based BT. Successful treatment of solid tumors using radio-NPs has been reported in several preclinical studies, on both mice and canine models. In this article, we review the recent advancements in the synthesis and use of radio-NPs as a substitute to seed-based BT. Here, we discuss the limitations of current seed-based BT and advantages of radio-NPs for BT applications. Recent progress on the types of radio-NPs, their features, synthesis methods, and delivery techniques are discussed. The last part of the review focuses on the currently used dosimetry protocols and studies on the dosimetry of nanobrachytherapy applications using radio-NPs. The current challenges and future research directions on the role of radio-NPs in BT treatments are also discussed.

## 1 Introduction

Cancer is one of the main causes of human death worldwide (1). Along with chemotherapy and surgery, radiotherapy (RT), also termed as radiation therapy, is a well-established method of treating non-metastatic cancers (2–4). In current practice, more than half of the cancer patients receive RT as primary mode of cancer therapy or adjuvant mode of treatment along with chemotherapy, immunotherapy, or surgery (5). In RT, high doses of ionizing radiation are delivered to ablate cancer cells and suppress recurrence and progression of cancer cells. RT can be broadly categorized into three types: external beam RT (EBRT), systemic RT, and internal RT (6, 7). In EBRT, high-energy photon or electron or ion beams are employed to deliver radiation to the tumor volume by placing radiation source outside the patient’s body (2). Systemic radiation therapies such as targeted RT deliver radioisotopes labeled with carrier molecules with high affinity towards receptors overexpressed by the cancer cells, e.g., monoclonal antibodies (mAb), through ingestion, infusion using catheter, or intravenous injection. In internal RT, also known as brachytherapy (BT), minimal invasive methods are used to place the radiation sources either inside or in close proximity to the tumor volume. BT allows delivery of high doses of radiation precisely to the tumor volume, while minimizing radiation exposure to the healthy tissues and organs at risk. Due to the precise and targeted dose delivery characteristics of BT, it can be employed to effectively treat solid tumors with minimum side effects and short treatment time at low cost.

Clinical trials and preclinical studies using BT have reported promising outcomes. However, the logistical and operational difficulties associated with BT seed placement have impeded its successful application. For instance, in patients with prostate tumor, the transrectal ultrasound (TRUS)-based implantation approach is used to implant radioactive seeds within the tumor (8, 9). The seed implantation causes trauma and edema in the prostate gland. This may consequently result in inaccurate or off-target placement of the seeds. The placement of radioactive seeds outside the tumor volume may result in undesired radiation exposure to the organs at risk, e.g., urinary bladder and rectum. Further, inaccurate seed placement may produce non-uniform dose distribution and may consequently result in mild to severe clinical side effects. Additionally, post-implantation migration of seeds to the lungs has also been reported and may require seed removal (8, 10).

Recently, several preclinical studies on localized delivery of radioactive nanoparticles (radio-NPs) into the tumor, similar to BT, have been reported in the literature, and this technique is termed as nanobrachytherapy (11–13). In nanobrachytherapy, radio-NPs are injected intratumorally as an alternative to the implantation of radioactive seeds. One recent example of this mode of treatment is the work by Salvanou et al. (14), who reported the use of gold nanoparticles (AuNPs) radiolabeled with ^225^Ac (alpha emitter) as an unconventional BT procedure, involving intratumoral injection of these radiolabeled AuNPs. Such nanoparticle-based systems i) conserve the characteristics of BT, i.e., precise and targeted dose delivery; ii) can be administered through injection; and iii) have the ability to provide patient-specific treatment, as radiation dose can be divided into several fractions. Additionally, these radiopharmaceuticals do not need seed removal; hence, they can be handled easily and can be extremely useful. The nanometer size of these radiopharmaceuticals allows local diffusion from the site of injection and may result in homogeneous dose distribution within the tumor volume. Lastly, these nanomaterials (particularly high Z nanoparticles) can be used as multifunctional carriers to deliver radioisotopes to provide imaging and RT capabilities. Such radioactive high-Z nanoparticles may also enhance radiation dose through self-sensitization and may require less radioactivity in comparison with conventional BT.

In this article, we review the recent advancements in the synthesis and use of radio-NPs as nanobrachytherapeutic agents. The subsequent section presents a review and discussion on different techniques involved in radiosynthesis of nanoparticles. The particles emitted by radionuclides, present in the obtained radionuclide–nanoparticle complex, must deposit their energy locally and spare the surrounding normal tissues. Hence, in the succeeding section, the essential characteristics of radionuclide–nanoparticle complexes, which are vital to qualifying them as nanobrachytherapeutic agents, are discussed. After intratumoral injection, these radio-NPs diffuse 1–2 mm within the extracellular medium, from the site of injection (15), and are internalized by the tumor cells. Thus, different mechanisms involved in the internalization of radio-NPs by tumor cells are reviewed in the next section. Thereafter, we summarize and review the most recently published preclinical studies on nanobrachytherapy. Additionally, for any RT-based treatment, dosimetry and treatment planning are the two crucial steps to ensure and quantify its accuracy and efficacy. Hence, the subsequent section reviews the recent dosimetric studies on use of radio-NPs as nanobrachytherapeutic agents. Lastly, current challenges and future research directions on the role of radio-NPs in BT treatments are discussed.

## 2 Methods of Radiosynthesis of Nanoparticles

Although several advances in cancer treatment have been made throughout the years, it is paramount to develop more precise diagnostic and therapeutic regimens essential to achieve better diagnostic and therapeutic outcomes. Tumor presents a multifactorial etiology, which makes it an extremely complex and heterogeneous disease, attributed to an almost unique expression of biomarkers from patient to patient. To circumvent this complexity, the development of so-called precision and personalized medicine is pivotal towards the battle against cancer (16). One of the major strategies is through the combination of nuclear medicine modalities and nanotechnology to offer unique opportunities to develop an effective single chemical entity with diagnostic and therapeutic capabilities for clinical applications in theranostic nanoradiopharmaceuticals. This is achieved by designing architectural radiolabeled nanoconstructs based on the amalgamation of four major components for the intended *in vivo* pharmacokinetics (17):

1. Appropriate nanoparticles including inorganic, organic polymers, and metallics
2. Targeting ligand (e.g., biomolecule, antibody, and peptide)—allows for specific targeting of receptors overexpressed on tumor cells or within the tumor microenvironment
3. Radionuclide selection (imaging and/or therapeutic)—emission mode, decay half-life, and chemical properties, availability, and radiolabeling reaction
4. Radiolabeling strategy to achieve the maximal radiochemical purity and yield, which reflects specific activity of nanoradiopharmaceuticals

Among the different types of nanoparticles, AuNPs and iron oxide nanoparticles (IONPs) have gained more prominence due to their superior biocompatibility, low toxicity, ease in surface versatile functionalization and radiolabeling with a plethora of imaging, and therapeutic radionuclides towards the development of nanoradiopharmaceuticals for imaging and therapy of cancer. Translational medicine that makes use of nanoradio-pharmaceutical agents demonstrates excellent pharmacokinetics in terms of radiochemical production, purity and stability (nanoradioformulation integrity), biodistribution, dosimetry, low off-target localization, and favorable renal clearance profiles, which represent a versatile theranostic tool in cancer management, ranging from nuclear medicine imaging and image-guided surgery to alpha/beta-particle targeted therapy, and most recently targeted nanobrachytherapy (18–21). The use of targeted nanobrachytherapy through radiolabeled nanoparticles affords intra- or peritumoral administration, thus allowing less invasiveness and homogenizing the radiation dose deposition in the tumor as compared with conventional BT (22).

### 2.1 Radiolabeling Nanoparticles

**Figure 1:**
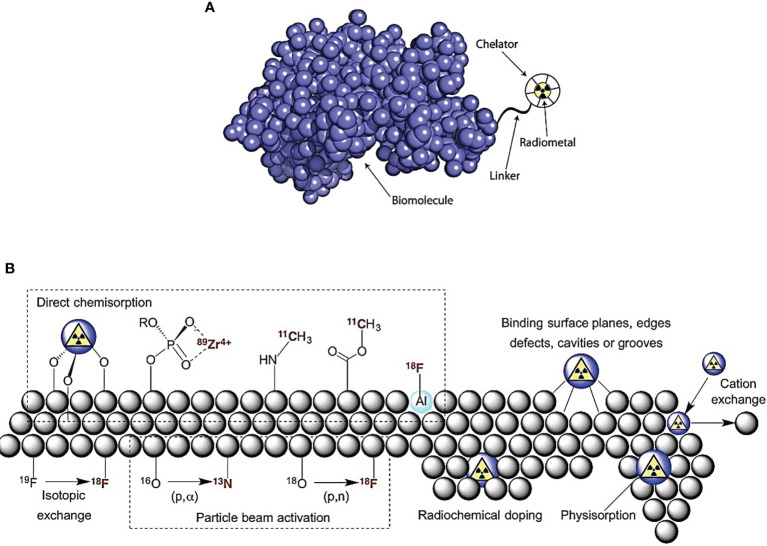
Radiolabeling nanoparticle strategies include the following: **(A)** indirect radiolabeling by bifunctional chelator—compounds having reactive functional groups that enable them to be covalently linked (conjugated) to biologically relevant vectors (e.g., protein and peptide). **(B)** Direct radiolabeling *via* i) chemisorption, high binding affinity chemical bonding between nanoparticles and radionuclides; ii) cavity entrapment, entrapment of radionuclides in native cavities or core-shell/layered nanoparticles; iii) isotopic exchange, exchanging stable and radioactive isotopes of an element in different chemical states; iv) particle beam activation, hadronic bombardment to initiate a nuclear reaction that converts stable isotopes in the nanoparticle lattice into radioactive nanoparticles; v) radiochemical doping, using a radionuclide as a surrogate during the synthesis, yielding inherently radioactive nanoparticles; vi) physisorption, physical bonding to the surface of nanoparticles by Van der Waals forces; vii) cation exchange, cation exchange between the nanoparticle**’**s cation and a different cationic radionuclide [adapted with permission from Lamb et al. (23)].

A plethora of orthogonal (radio)labeling strategies for nanoparticles are available for the development of multimodal nanoradiotherapeutics (23) as shown in **Figure 1**. The radiolabeling of nanoparticles for medical imaging and therapy has been discussed in-depth in reviews, which are highly recommended for further reading (20). The most pertinent consideration for radiolabeling nanoparticles is the functionalization with suitable molecular entities to allow for the coordination/conjugation of the radioisotopes achieved through the use of chelators *via* coordination chemistry approaches (19, 20, 24):

1. Bifunctional moieties that provide capping/stabilizing capabilities with subsequent binding affinity to the radioisotopes
2. Direct surface conjugation of amino/thiolated molecules followed by ligand exchange
3. Chemical modification of molecules already attached on the surface of the nanoparticles

#### 2.1.1 Indirect Radiolabeling

Indirect radiolabeling is attainable *via* exogenous coordination chemistry moieties [bifunctional chelators (BFCs) and prosthetic groups] through chemical linkers to aid complexation (25).

##### 2.1.1.1 Bifunctional Chelators

BFCs are molecules consisting of a metal chelating unit that binds to metallic radionuclides and a reactive functionality for conjugation with surface of the nanoparticles. BFCs are highly preferred due to *in vivo* radiolabel stability strongly dependent on the coordination chemistry between the radionuclide and the BFC. However, the drawback of radionuclide–BFC coordination complexes is *in vivo* dissociation due to enzymatic and/or trans-chelating interactions with proteins such as transferrin and ferritin. A successful BFC allows for minimal *in vivo* dissociation of the radionuclide from the chelator, dependent on the kinetic inertness and thermodynamic stability of the BFC, where polydentate ligands form stable complexes over their monodentate ligands due to the “chelate effect” (19, 20). The bioconjugation of BFC to nanoparticles is usually facilitated by functional groups present on the surface of nanoparticles that include amine conjugation (e.g., anhydride, NHS ester, and isothiocyanate), carboxylic acid conjugation (e.g., carbodiimide couplings), thiol conjugation (e.g., maleimide coupling), and click chemistry conjugation (e.g., Cu-catalyzed azide-alkyne cycloaddition and inverse electron demand Diels–Alder cycloaddition) to ensure the *in vivo* inertness of the resulting radiometal complex (20). The chelator selection is dependent on the radionuclide and desired physicochemical properties and pharmacokinetics of the radiolabeled nanoparticles.

**Figure 2:**
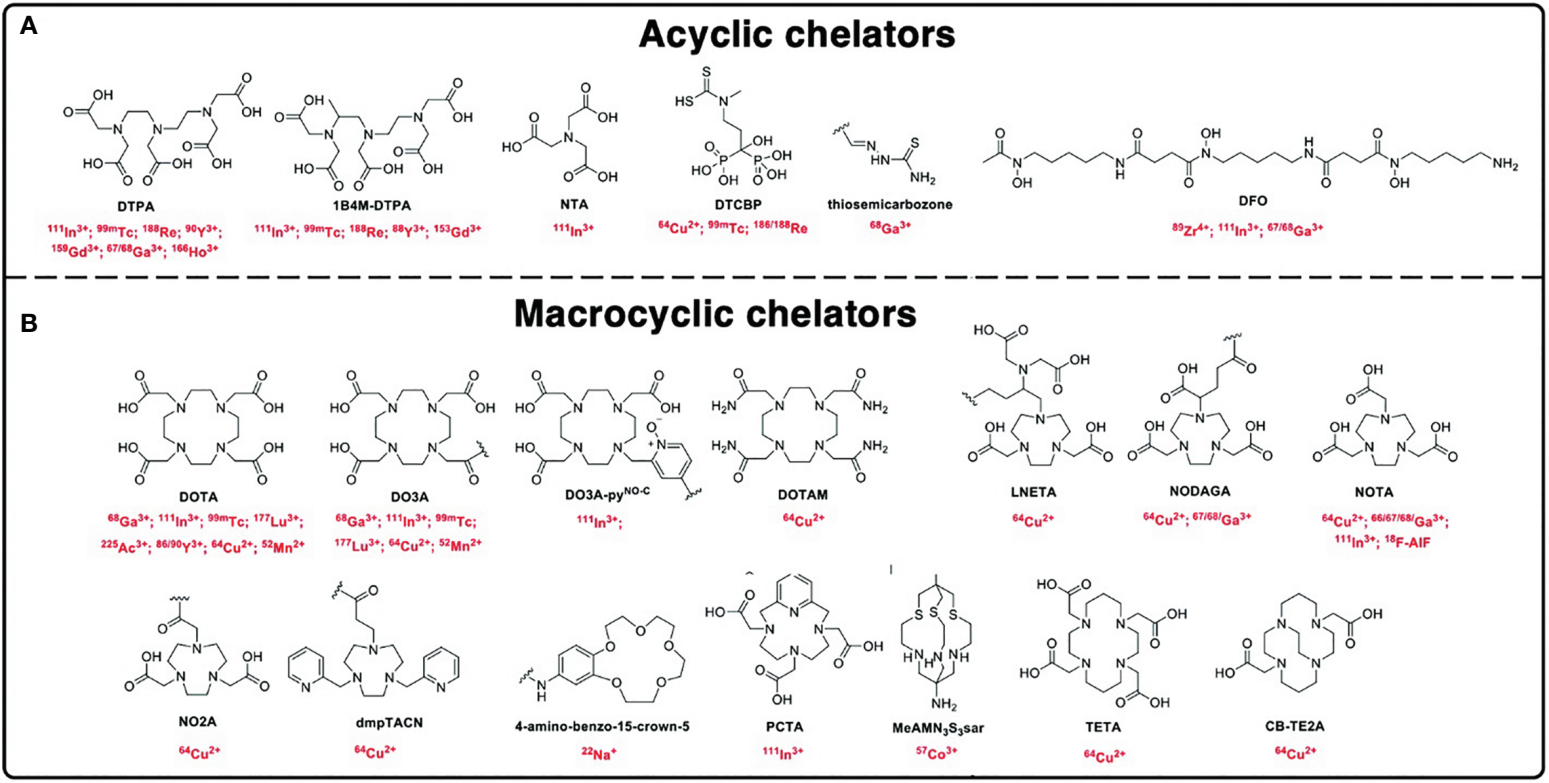
Chemical structures of the chelators. **(A)** Acyclic chelators and **(B)** macrocyclic chelators and their respective radionuclides used for radiolabeling nanomaterials [adapted from Pellico et al. (20)].

Categories of BFCs (**Figure 2**):

1. Macrocyclic chelators—relatively rigid and pre-organized structure allowing for high complexation stability due to macrocyclic effect but suffer from slow complexation kinetics
2. Acyclic/linear chelators—offer rapid radiometal complexation due to their lack of rigidity

###### 2.1.1.1.1 Radiolabeling via Dodecane Tetraacetic Acid-Based Chelators

Macrocyclic multidentate chelator, dodecane tetraacetic acid (DOTA), is the most commonly utilized BFC owing to its high affinity to most metal radionuclides (^64^Cu, ^177^Lu, ^68^Ga, and ^111^ln). Among the radionuclides, ^177^Lu (*t*_1/2_ = 6.734 days) with both *β* emissions and *γ* rays is of interest for theranostics. ^177^Lu entrapping AuNPs inside the dendritic cavity of a generation 4 (G4) polyamidoamine (PAMAM) dendrimer, which had been pre-conjugated with *p*-SCN-benzyl-DOTA as well as folate/bombesin for cancer targeting (26). Cancer immunotherapy with mAb such as atezolizumab, pembrolizumab, and trastuzumab has been conjugated to DOTA and radiolabeled with ^64^Cu (^64^Cu-DOTA-mAb) for positron emission tomography (PET) imaging utilized to estimate tumor density, perfusion, and distribution in mice bearing MDA-MB231 anti-programmed death-ligand 1 (PD-L1-positive) xenograft and HER2-targeted antibodies for patients with metastatic HER2-positive breast cancer (BC) (27, 28). Poly-(isobutylene-alt-maleic anhydride)-graft-dodecyl (PMA) is a polymer shell, which was integrated with DOTA for ^111^ln loading, thus resulting in ^111^ln-DOTA/^198^Au nanoparticles being classified as a post-formulation chelation (21). Hajiramezanali et al. (29) developed ^68^GA-radiolabeled bombesin conjugated to trimethyl chitosan-coated superparamagnetic nanoparticles (^68^Ga-DOTA-BN-TMC-MNPs) with radiochemical purity >98%. Most recently, AGuIX^®^ represents gadolinium (^67^Gd)-DOTAGA cyclic chelates covalently grafted to polysiloxane matrix to produce AGuIX nanoparticles (30, 31).

###### 2.1.1.1.2 Radiolabeling via 1,4,7-Triazacyclononane-N,N′,N″-Triacetic Acid-Based Chelators

A hexadentate N_3_O_3_ chelator, 1,4,7-triazacyclononane-*N*,*N*′,*N*″-triacetic acid (NOTA), and its derivative are commonly used for gallium and copper radiopharmaceuticals (^67^Ga/^68^Ga and ^64^Cu) for radiolabeling nanoparticles. The general approach for conjugating nanoparticles with NOTA moiety for ^67^/^68^Ga and ^64^Cu labeling is through thiol-functionalized NOTA (NOTA-SH) for radiolabeling and conjugation, additionally linkers/spacers such as polyethylene glycol (PEG) and PEI to optimize *in vivo* pharmacokinetics. NOTA-SH can be achieved by reacting *p*-SCN-Bn-NOTA with 2-aminoethanethiol hydrochloride in the presence of triethanolamine.

###### 2.1.1.1.3 Radiolabeling via Diethylenetriaminepentaacetic Acid-Based Chelators

A polydentate acyclic chelator, diethylenetria-minepentaacetic acid (DTPA), is commonly used in the construction of MRI and nuclear imaging agents (^99m^Tc, ^111^ln, and ^67^/^68^Ga). However, the DTPA complex exhibits low *in vivo* kinetic stability characterized by fast dissociation kinetics and radiometal complexation, and the functionalization of nanoparticles with polymers such as PAMAM and PEI improved stability.

##### 2.1.1.2 Prosthetic Groups

Indirect radiolabeling *via* chelators is susceptible to *in vivo* radiometal trans-chelation with native biological chelators and ions as well as metalloenzymes, transport, and storage proteins in the body. This problem is evaded by radiolabeling with non-metallic radionuclides covalently bound to nanoparticles through prosthetic groups (^11^C, ^14^C, ^18^F, ^123^I, ^124^I, ^125^I, and ^131^I) (25). [^18^F]-Fluoro-2-deoxy-d-glucose (^18^F-FDG) is used for the assessment of glycolysis as a non-invasive PET imaging agent. In an archetypical example, radiolabeling nanoparticles with ^18^F has been reported by first conjugating cysteamine to mannose triflate (Man-CA) and then ^18^F labeling resulting to a cysteamine-linked radiotracer (^18^F-FDG-CA). The ^18^F-FDG-CA is mixed with gold chloride (HAuCl_4_) to obtain AuNPs (^18^F-FDG-CA-AuNPs) (32).

##### 2.1.1.3 Ionophore-Based

Ionophore-based radiolabeling is divided into subclasses: i) ionophore-chelate binding and ii) remote loading radiolabeling. Both ionophore-chelate binding and remote loading radiolabeling use lipophilic radiotracers with passive lipid membrane permeability properties (20). Radiolabeling based on ionophore ligand binding to radionuclide metal ion through lipophilic radio-ionophore complexation allows for transport across lipid bilayers. Once internalized in the vesicle, the radiometal dissociates from the radio-ionophore complex and binds to chelating molecules (e.g., proteins/nucleic acids or drugs) within the vesicle, which is preferentially relevant for vesicle-based nanoparticles such as liposomes and exosomes/extracellular vesicles containing lipid bilayer membranes. Remote loading is similar to ionophore-based chelator with the addition that the complex contains functional groups that can be charged within the vesicle core. Aranda-Lara et al. (33) reviewed the radiolabeling of liposomes and lipoproteins as lipidic nanoparticles.

#### 2.1.2 Direct Radiolabeling

Indirect radiolabeling using chelator-based (bifunctional and prosthetic group) has gained prevalence in nuclear medicine. The negative impact on the biological activity of the overall radiolabeled nanoparticles is attributed to changes in the size, surface charge, and hydrophilicity of the nanoparticles. This problem can be overcome through direct and chelator-free radiolabeling strategies while maintaining the nanoparticle’s native pharmacokinetic characteristics.

##### 2.1.2.1 Chemisorption and Physisorption

Chemisorption is facilitated by mixing the radionuclides with nanoparticles that exhibit high binding affinity towards the radionuclides for direct chemical bond formation between the surface of the nanoparticles and the radionuclide. This is achieved through the oppositely charged moieties on the surface of the nanoparticles and the radionuclide, thus allowing for chemical adsorption. Likewise, physisorption occurs when charged radionuclide ions interact with the molecular surface of the nanoparticles *via* electrostatic attraction or van der Waals interactions (23). Pei et al. (34) designed a simple chelation between glutathione-modified gold nanoclusters (AuNCs) and radionuclides (^99m^Tc and ^177^Lu) to produce ^99m^Tc@AuNCs and ^177^Lu@AuNCs, respectively, as a novel approach for tumor radio-immunotherapy.

##### 2.1.2.2 Radiochemical Doping (Hot-Plus-Cold Precursors)

Radiochemical labeling involves incorporation of the radionuclide as a surrogate during the synthesis of the nanoparticles resulting in intrinsically radioactive nanoparticles often carried out in automated closed lead-shielded unit due to the increased radiation exposure (21, 32). This type of radiolabeling is divided into two subcategories: hetero-radionuclides, where nanoparticle core cation and the radionuclide are different (e.g., doping AuNPs with ^64^Cu or ^111^ln), and homo-radionuclides, where a radioisotope of the metal element to form the nanoparticle core is used (e.g., premixture of H^198^AuCl_4_ to HAuCl_4_ precursor for the production of ^198^AuNPS) (10, 35, 36). Similar studies by Laprise-Pelletier et al. (15) produced ^103^Pd : Pd@^198^Au : Au-PEG nanoparticles by premixing ^103^PdCl_2_/PdCl_2_ and H^198^AuCl_4_/HAuCl_4_; and Chakravarty et al. (37) produced ^199^Au nanoparticles conjugated with cyclic arginine-glycine-aspartate peptide (^199^AuNP-RGD) by intrinsically radiolabeling during synthesis of AuNPs through the use of H^199^AuCl_4_ precursor. Fach et al. (38) doped [^103^Pd]PdCl_2_ in a solution of HAuCl_4_ for co-reduction to produce AuPdNPs intrinsically labeled with ^103^Pd ([^103^Pd]AuPdNPs) with ≈20 nm, and then ethylenediaminetetraacetic acid (EDTA) was used to scavenge free Pd^2+^ to avoid unspecific labeling of the nanoparticle surface resulting in radiolabeling efficiencies of 79% to >99%.

##### 2.1.2.3 Hadronic Bombardment (Particle Beam Transmutation/Activation)

Formulated nanoparticles/nanocarriers contain stable precursors of the desired radionuclide (21). Radiolabeling *via* hadronic bombardment is performed by irradiating prefabricated nanoparticles *via* bombardment with accelerated particles (i.e., neutrons, protons, or deuterons) using a high-energy particle accelerator or nuclear reactor to induce a nuclear reaction to convert the stable isotope in the nanoparticle lattice to radioisotopes, resulting in radio-NPs. This radiolabeling is controlled by the bombardment time, current, and beam-line energy; the latter energies are often >10 MeV higher than for nanoparticle stability. To overcome this, an effective heat dissipation technique is a prerequisite for this method. Pérez-Campaña et al. (39) produced [^13^N]Al_2_O_3_NPs by 16-MeV proton irradiation of Al_2_O_3_NPs *via* the 16O(p,α) ^13^N nuclear reaction.

##### 2.1.2.4 Encapsulation (Cavity Entrapment)

Encapsulation is through entrapping the radionuclide inside the native cavity within the nanoparticles or within core-shell/layered structured nanoparticles. Lee et al. (40) demonstrated the encapsulation of ^124^I or ^125^I to produce ^124^/^125^I embedded AuNPs. This was achieved by modifying the amine groups of the adenine-rich oligonucleotides on the surface of the AuNPs with sulfosuccimidyl-3-[4-hydroxyphenyl]propionate for ^124^I or ^125^I radiolabeling, followed by reacting the nanoparticles with HAuCl_4_ to form a Au shell to shield radionuclide dissociation, thus resulting in ^124^/^125^I-Au@AuNPs this approach was further used to produce ^124^I-labeled tannic acid gold core-shell nanoparticles (^124^I-TA-Au@AuNPs) exhibiting 98% radiochemical yield. Laan et al. (41) reported a facile method for ^111^ln-labeling polystyrene-*b*-poly (ethylene oxide) diblock copolymer micelles without the necessity of any chemical modification.

### 2.2 Heterogeneous/Homogeneous Radioisotopic Exchange or Cation Exchange

#### 2.2.1 Heterogeneous/Homogeneous Radioisotopic Exchange

Isotope exchange is facilitated through chemical equivalent exchange between the stable and radioactive isotopes of an element in different chemical states resulting only in low specific activity. For example, Freund et al. (42) produced ^59^Fe-labeled IONPs by oleic acid-functionalized IONPs in chloroform, and then the IONPs were incubated with ^59^FeCl_3_ which led to approximately 0.01%–0.5% ^59^Fe exchange with Fe^3+^ (homogenous) in the IONPs. The low isotope exchange of ^59^Fe/Fe is attributed to Fe surface availability of the IONPs. Heterogeneous radioisotopic exchange was demonstrated by Tang et al. (43) chelator-free radiolabeling of zinc sulfide (ZnS) quantum dots (QDs) with ^68^Ga or ^64^Cu through cation exchange.

#### 2.2.2 Cation Exchange (Radio-Halogenation)

Similar to isotope exchange approach, cation exchange is a relatively new alternative that is more effective but still needs some improvements. It is carried out by a cation exchange between the cation within the nanoparticle and a different cationic radionuclide. Gaikwad et al. (44) intrinsically radiolabeled chitosan nanoparticles with ^177^Lu *via* ionic gelation technique to produce ^177^Lu-labeled chitosan nanoparticles (^177^Lu-CH NP) with >98% radiochemical purity. Zhang et al. (45) developed PEGylated covalent organic frameworks (COFs) with strong affinity for Ag^+^ ions, followed by ^125^I radiolabeling at the Ag site to produce nanoscale ^125^I-labeled PEG-COF-Ag with 94% radiolabeling yield in 30 s for BT.

In conclusion, beta emitters are preferred radionuclides over their alpha counterparts during radiolabeling owing to the large recoil energy (in the order of 100 keV) during decay of the latter (46). However, targeted alpha therapy (TAT) has received sufficient attention; therefore, effective radiolabeling strategies have been developed. Recently, Yi et al. (47) developed X-ray-optimized delivery of radiolabeled albumin for cancer theranostics. The authors utilized the abundant tyrosine existing in human serum albumin (HSA) nanoparticles for ^125^I/^131^I radiolabeling forming iodotyrosine for the production of ^125^I/^131^I-HSANPs.

## 3 Radionuclides for Nanobrachytherapy

The radionuclides to be used for internal RT must deliver high doses of radiation locally and spare the surrounding normal tissues (5, 13). Hence, radionuclides emitting radiation with higher linear energy transfer (LET) are generally preferred. LET is the amount of energy transferred, by the emitted particles, to the medium traversed per unit distance. These radionuclides are categorized into three groups based on the emitted particle type (48). It includes *α*, *β*, and Auger particle-emitting radionuclides, as reported in **Table 1**.

**Table 1:** Summary of radionuclides and radioactive nanocarriers investigated in preclinical studies on nanobrachytherapy.

Radioisotopes	Half-life [days]	Decay mode	Emissions	Energy [keV]	Range max	References
			*β*	961 (99%), 285 (1%)	4 mm	
Au-198	22.7	*β* (100%)	*γ*	412 (96%), 676 (<1%), 1088 (<1%)	–	(49)
			*β*	462 (6.0%), 296 (71.6%), 250 (22.4%)	–	
Au-199	23.1	*β* (100%)	*γ*	159 (37%)	–	(37)
			*β*	497 (79%), 385 (9%)	1.6 mm	
Lu-177	26.7	*β* (100%)	*γ*	208 (11%), 113 (6%)	–	(50)
			*β*	248 (2%), 334 (7%), 606 (90%)	0.6 mm	
I-131	28	*β* (100%)	*γ*	284 (6%), 365 (82%), 637 (7%)	–	(17)
Ac-225	10	*α* (100%)	*α*	5800 (100%)	100 um	(14)
At-211	0.3	*α* (100%)	*α*	5870 (100%)	–	(51)
Pd-103	17	EC (100%)	*γ*	20 (64%), 23 (13%)	–	(38)
I-125	59.9	EC (100%)	*γ*	27 (114%), 31 (26%), 36 (7%)	–	(45)
In-111	2.8	EC (100%)	*γ*	245 (94%), 171 (90%)	–	(52)

EC, electron capture.

It is important to evaluate the suitability of these radionuclides for nanobrachytherapy applications. The must-have features for radionuclides can be classified into two main groups: i) physical and ii) biochemical characteristics. The physical characteristics to be considered are a) physical half-life; b) emitted particle type—*α*, *β*, and Auger electrons or photons; c) energy of the emitted particles; d) daughter product(s) and their stability; e) radionuclide purity and length of purification step; f) penetration depth of the emitted particles in the biological tissues; g) LET of the emitted particle; and h) size of the tumor to be treated (5, 13). Additionally, the biochemical characteristics to be evaluated are a) approach used to target tumor cells/tissues; b) retention of radio-NPs within the tumor; c) *in vivo* stability of the radionuclide–nanoparticle complex; and d) toxicity caused by the complex (53–55).

The physical half-life of the radionuclide should match with the *in vivo* pharmacokinetics of the radionuclide–nanoparticle complex (55). The life span (T) of the radionuclide can be estimated from the physical half-life (*Tp*) of the radionuclide and half-clearance time, also known as biological half-life (*T_b_*), of the radionuclide–nanoparticle complex using the relation 1/T = 1/*T_p_* + 1/*T_b_* (56). The physical half-life of the radionuclide can be known from the published radionuclide data; and to estimate the biological half-life of the radionuclide–nanoparticle complex, knowledge on the spatial and temporal distribution of the complex within the tumor and body is required. *T_b_* depends on the mode of delivery, uptake, and metabolism of the radionuclide–nanoparticle complex by the tumor cells and its excretion from the patient’s body (48, 53, 54).

The radionuclides with physical half-life of between 6 h and 7 days are preferred for therapeutic purposes. An extremely short physical half-life hampers the flexibility in administration of the radiotherapeutic agent and is impractical for clinical use. On the contrary, the use of long-lived radionuclides may result in retention of radiation dose in the patient for a longer period of time. Furthermore, patients may be required to be isolated and admitted in the hospital, in order to minimize the risk of radiation exposure to the general public. Additionally, the biological half-life of the radionuclide–nanoparticle complex is dependent on the properties of the nanocarrier used. The nanocarriers with long biological half-life should be used with radionuclides having short physical half-life (13, 48). The radio-NPs must be efficiently retained within the tumor volume so that higher doses of radiation can be delivered to the tumor tissues. The use of nanocarriers with short biological half-life may result in excretion of radio-NPs with high activity and may need extensive management of radioactive waste. Hence, for efficient delivery of radiation dose, the radionuclide–nanoparticle complex with optimal physical and biological half-life must be selected (48, 54, 55).

α-Particle emitters such as ^225^Ac and ^211^At emit positively charged helium nuclei, having high higher LET and short penetration depths in biological tissues (5). For instance, ^225^Ac emits alpha particles in an energy range of 5–9 MeV and has LET value between 80 and 100 keV/μm and spatial penetration range between 40 and 100 μm. Hence, it has a probability of depositing most of the radiation within the tumor volume and can ablate tumor cells efficiently. Thus, α-emitting radionuclides are suitable in treating small or residual microscopic-size tumors. The main limitation of α-emitting radionuclides is that they have multiple daughter products with variable half-lives. Hence, migration of these nanocarriers labeled with α emitters can lead to significant damage to normal tissues (13, 14, 51, 57).

β-Emitting radionuclides are the most widely used radionuclides for internal RT purposes. The emitted electrons have lower LET and longer range (several millimeters) in comparison with the α emitters (58, 59). For example, ^90^Y emits electrons with LET of 0.2 keV/μm and mean range of 3,960 μm. Hence, it may result in less cytotoxicity in comparison with the α-emitting radionuclides and radiation damage caused by these long-range β-particles, far from its origin, which is termed as “crossfire effect.” Thus, due to the long penetration depth of the emitted electrons (≈0.05–12 mm), β emitters are regarded as the most suitable for the treatment of large or bulky tumors (7). β-Emitting radionuclides ^198^Au, ^199^Au, ^131^I, and ^177^Lu have been investigated as potential nanobrachytherapeutic agents (17, 37, 49, 50). ^198^Au was used in the initial works of radioactive collidal gold (60). It is because ^198^Au can be easily integrated with AuNPs. Some β-emitting radionuclides also decay with γ-radiation. For nanocarriers composed of high-Z materials, AuNP in particular, gamma radiation on interaction with the material of the nanoparticles may result in the enhancement of radiation dose deposition by the mechanism of radiosensitization (13, 15). Photoelectric effect plays a vital role in radiosensitization, and for Au, it is the strongest for gamma radiation of energy below or equal to 200 keV. ^198^Au, ^131^I, and ^177^Lu emit gamma radiation with energy >200 keV. Hence, the photoelectric effect for gold is the strongest for photons with energy lower than 200 keV. The gamma radiation emitted by these radionuclides does not provide maximum radiosensitization effect (5). However, ^199^Au emits gamma radiations with maximum energy ≈158 keV. Thus, dose enhancement *via* radiosensitization effect can be expected. In this regard, gold was used as a nanomaterial in preclinical studies, using AuNPs radiolabeled with β-emitting radionuclides, due to its biocompatibility. Additionally, this gamma emission associated with β-emitting radionuclides can be advantageous in visualizing the spatial and temporal distribution of radio-NPs within the patient with the help of gamma scintigraphy techniques. Lastly, it should be considered that the long range of emitted electrons may result in non-specific cytotoxicity by depositing radiation dose to the surrounding normal cells/tissues (48, 54, 55).

Radionuclides emitting Auger electrons are considered to be beneficial in the treatment of small tumors or a cluster of tumor cells. This is attributed to higher cytotoxicity caused by these low-energy electrons (less than 500 eV or a few keV) with short range in the biological tissues (a few nanometers) (5, 54, 55). ^103^Pd, ^111^In, and ^125^I have been used as nanobrachytherapeutic agents in preclinical studies involving tumor-bearing xenograft models (38, 45, 52). These radionuclides decay by internal conversion (IC) and electron capture (EC) mode and emit Auger electrons. The energy of the emitted Auger electrons range from ≈500 eV to a few keV with a spatial penetration depth of 2–500 nm. For effective ablation of tumor cells, these radionuclides must be internalized as close as possible to the cell nucleus. These radionuclides, ^103^Pd, ^111^In, and ^125^I, also emit gamma radiation. ^125^I and ^103^Pd emit low-energy (30 keV) photons and have been used for low-dose-rate BT applications since the 1970s. The emitted photons deposit up to 98% of their energy within ≈5–8 cm of soft tissue and can be used to treat large and bulky tumors. ^111^In also emits photons with energy greater than 200 keV and is not suitable for internal RT purposes or radiation dose enhancement through radiosensitization (13). In case of preclinical studies using xenograft models, the energy deposited to the tumor models is mainly due to these emitted Auger electrons and photoelectrons generated due to the interaction of low-energy photons and gold (7, 61).

Hence, the choice of the radionuclide also depends on the size of the tumor to be treated. It is because bulky tumors, micrometastases, and a small cluster of tumor cells require particles of specific energy for effective ablation of cancer cells. Further, the mode of radiosynthesis of nanoparticles and the length of the purification step (of radionuclides) must be selected according to the half-life of the radionuclide (54, 55). In terms of the spatial penetration depth and energy of the emitted particles, Auger and β-emitting radionuclides are most suitable for the treatment of solid tumors such as brain, breast, and prostate tumors by using nanobrachytherapy procedures (5, 13, 61).

Considering biochemical properties, a clinically acceptable radionuclide–nanoparticle complex must selectively concentrate within the tumor and have a prolonged retention. Also, it should have minimum or no uptake in the normal tissues or organs. Furthermore, the ratio of retention of a nanobrachytherapy agent should be high in the tumor volume in comparison with the normal tissues (10), so that high radiation doses can be delivered to the tumor volume and minimum or no radiation dose is delivered to the normal tissues or organs. Additionally, the radionuclide–nanoparticle complex should be stable enough at the time of injection and should have prolonged retention *in vivo* before it is excreted or metabolized (5, 13). Other biochemical features to be taken care of are low toxicity, appropriate pH, and optimal biological half-life. Furthermore, the radionuclide and nanoparticle (to which a radionuclide is attached) must have a high complexation yield and must form a stable complex in the biological environment (48, 53).

## 4 Mechanisms for Nanoparticle Internalization

**Figure 3:**
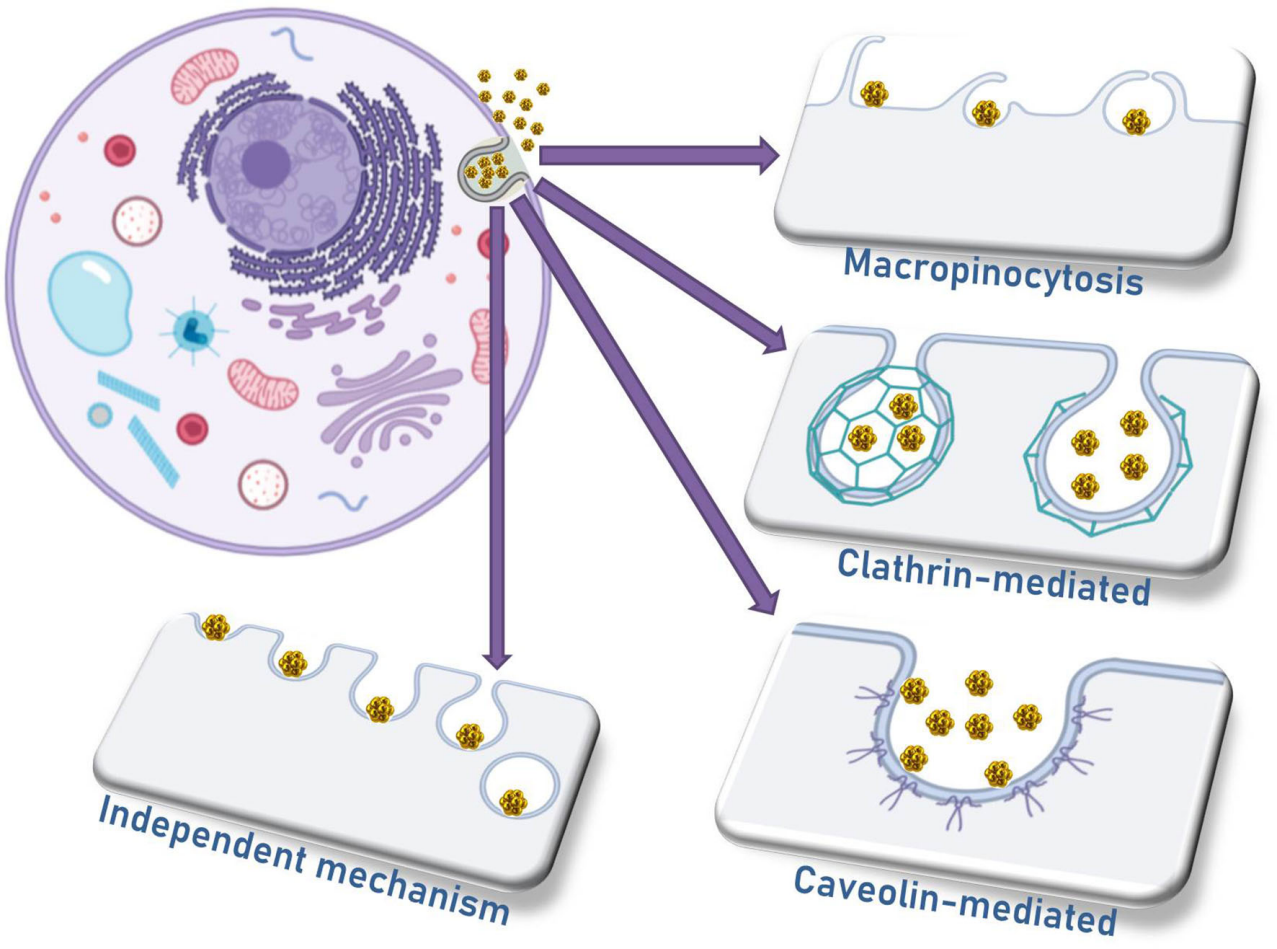
The four main mechanisms responsible for the cell internalization of nanoparticles.

The four main mechanisms of nanomaterial internalization by cells are micropinocytosis, caveolae-mediated endocytosis, clathrin-mediated endocytosis, and a mechanism independent of caveolae or clathrin (**Figure 3**) (62). The differential profile of AuNP internalization by different cell types depends on a large extent to the differences in their biophysical mechanisms, especially the cell membrane characteristics. Regarding the nanoparticle characteristics, the uptake is influenced significantly by the surface chemistry and the morphology of different nanomaterials. Additionally, one should be aware of the size of nanoparticle clusters that might be formed by aggregated particles in contact with cells, and the consequence of this aggregation in the internalization efficiency, as well as the location of nanoparticles and nanoaggregates in terms of organelles and intracellular vesicles (63, 64).

In 2008, Douglas and colleagues investigated the internalization and cytotoxicity of alginate–chitosan nanoparticles in 293T, COS7, and CHO cells. It was demonstrated that trypsinization can prevent alginate–chitosan nanoparticle internalization depending on the cell type. After trypsinization in 293T and COS7 cells, 75–85% of the binding efficiency to plasma membrane was lost, indicating that the interaction of those nanoparticles with the cells was mediated by chitosan and trypsin-sensitive proteins, but the same was not observed in CHO cells (65).

In the same study, it was observed that the vectors were not localized in lysosomes once they enter the cells, and the endocytic mechanism is different among the studied cell lines. For instance, clathrin-dependent endocytosis is important in 293T and COS7 cell lines, while caveolin-dependent internalization is significant for COS7 and CHO cells. Macropinocytosis was not relevant for any of the cell lines, but another mechanism dependent on actin microfilaments plays an important role for the internalization in 293T cells. This study supported the assumption that many factors are important for cell internalization and for the fate of nanomaterials in the cells, i.e., cell physiology, complex size, composition, and endocytosis mechanism. These parameters must be fully indicated in order to increase the success rate in the designed treatment (65).

The mechanism of internalization of 200-nm-diameter nanoparticles seems to be a combination of energy-dependent phagocytosis and clathrin-mediated endocytosis. But in all cases, the endocytoses were proven to be energy-dependent, while for smaller particles, an actin-dependent mechanism seems to play an important role. Caveolae-mediated endocytosis is the most important mechanism for 150- and 200-nm nanoparticles, but it is worthy to mention that all internalization pathways contribute to the internalization of 150-nm nanoparticles, and this might explain the higher efficiency of endocytosis for those particles (66). Positively charged nanoparticles were observed to be significantly more internalized than negatively charged ones (84% against 5%, respectively). It is clear that negatively charged particles must rely on surface functionalization so that receptor-mediated endocytosis can compensate for the lower internalization rates.

Bannunah and collaborators published a thorough study comparing negatively and positively charged particles, of different sizes, in terms of their epithelial and cell uptake efficiency, as well as their toxicity to CaCo-2 (human intestinal adenocarcinoma) and Calu-3 (human airway epithelial) cells. According to their study, positively charged nanoparticles cause higher levels of cytotoxicity as compared with negatively charged ones, and it might be due to the oxidative stress, mitochondrial damage, and cellular overall toxicity observed for those kinds of particles. Negatively charged particles are known to be less cytotoxic to epithelial cells, and this might be explained by the fact that those cells present a net negative charge in their extracellular portion of plasma membrane, enabling a better interaction with positively charged nanoparticles. The results obtained for other cell types are sometimes conflicting; therefore, more studies might be necessary in order to understand the mechanisms for each tissue (67).

When inhibitors of dynamin-dependent and clathrin-dependent endocytoses are used, it seems that both negatively and positively charged nanoparticles are not significantly internalized *via* a dynamin-dependent mechanism, but the inhibition of clathrin-mediated transport likely caused an increase in the transport of negatively charged particles, though with no effect on their cell uptake. Regarding positively charged particles, clathrin inhibition reduced by 46% their cell uptake and by 38% their transcellular transport, whereas micropinocytosis inhibition reduced the internalization of the same particles by 42%, and the transcellular transport by 38%, similarly to micropinocytosis inhibition by methyl-β-cyclodextrin (67). No effect on negatively charged nanoparticles was observed after micropinocytosis inhibition.

The disruption of microtubules with nocodazole had no effect on the internalization of any of the nanoparticles, but the transport across the cells was significantly impaired. Genistein, a tyrosine-kinase inhibitor, impaired both the internalization (50%) and the intracellular transport (48%) of negatively charged nanoparticles, leading to the assumption that caveolae-dependent endocytosis plays an important role for those nanoparticles (67).

The protein corona is another key factor to be considered when developing any nanomaterial with biomedical applications. The protein corona is formed whenever a nanomaterial is introduced into a complex protein aqueous system and consists in the rapid adsorption of the most abundant proteins onto the surface of the nanomaterial, followed by the exchange of at least part of these proteins for others with higher affinity for the nanomaterial. The result is a nanoparticle with a completely different surface coating than that predicted in the design phase, sometimes with tendency for aggregation or with higher stability, with different internalization rates (enhanced or impaired), and different pharmacokinetics (68, 69).

The composition of the protein corona is not universal, as it depends on the nanomaterial and on the previous coating. It was demonstrated, for instance, that citrate-coated IONPs were not stable while in contact with fetal calf serum proteins but were efficiently internalized by lymphoblastoid cells, while poly(acrylic acid)-coated IONPs were quite stable, although were poorly taken up, which can be a barrier to be faced by the nanomaterials inside the blood (68).

Another barrier that the nanoparticles must overcome is the reticuloendothelial system (RES), responsible for a rapid clearance of the nanomaterials once they enter in the bloodstream, decreasing their pharmacological action. Strategies to avoid the clearance by RES include surface modification with molecules that prevent opsonization and increase the half-life in blood, such as PEG. However, as described previously, many variables must be added, such as surface charge. Harush-Frenkel and collaborators verified once more the preferential internalization of positively charged nanoparticles (twice the endocytosis of their negatively charged counterparts) in HeLa cells, and after 45 min, the cells tend to decrease the uptake rate, characterizing a saturation phase (70).

Another factor that contributes to the decreased circulating time of nanoparticles in blood is the mononuclear phagocytic system, in which the macrophages quickly scavenge nanoparticles that are agglomerated or covered with the protein corona, preventing their arrival at the target site. Zhang and coworkers used the advantage of folic acid as a functionalizing agent in PEGylated superparamagnetic magnetite nanoparticles (circa 10 nm in diameter) in the internalization efficiency by mouse macrophages (RAW 264.7 cells) and human breast tumor (BT-20 cells). PEG was responsible in partially inhibiting the formation of the protein corona in order to decrease the recognition of nanoparticles by macrophages, whereas folic acid was added to the surface of nanoparticles to specifically target cancer cells overexpressing folate receptors in order to increase their uptake (71).

## 5 Preclinical Studies on Nanobrachytherapy

The alpha-, beta-, and Auger-emitting radionuclides have been investigated for nanobrachytherapy applications. A few preclinical studies on nanobrachytherapy applications using alpha, beta, and Auger emitters have been published in literature. The most recent ones are briefly discussed in this section, and a brief summary of these studies is also presented in **Table 2**.

**Table 2:** Summary of nanobrachytherapy-based preclinical studies.

Main type of emission	Nanoparticle	Study (tumor model) type	Target	Main results	Reference
α	AuNPs (5 and 15 nm) functionalized with a peptide from Substance P(5-11) and labeled with ^211^At.	*In vitro* (-).	NK1 receptors overexpressed in T98G glioma cells.	The authors recommended the intratumoral injection of the NPs instead of intravenous injection due to the their large size.	(51)
α	AuNPs (5 nm) with chemically adsorbed ^211^At and activated with PEG and trastuzumab.	*In vitro* (-).	HER-2 proteins overexpressed in SKOV-3 cell ovarian cancer cells.	AuNP-S-PEG-trastuzumab bioconjugate was effectively internalized by SKOV-3 cells and reduced the metabolic activity of ovarian cancer cells with a median lethal dose of 0.5 MBq/mL.	(57)
α	AuNPs (5–9 nm) radiolabeled with ^225^Ac using TADOTAGA chelator.	*In vivo* (U-87 MG tumor xenograft).	Nanobrachytherapy for xenograft bearing U-87 MG human glioblastoma–astrocytoma cells.	For mice (therapy group) injected with 100 μL/5 kBq of [^225^Ac]^225^Ac-Au@TADOTAGA per mouse (on days 1, 3, and 5), the tumor volume was reported to be ≈2.4 times lower after 8 days of radioactive injection and ≈4 times lower after 22 days of injection, in comparison with the control group.	(14)
Auger electrons	AuNPs coated with a layer of ^103^Pd (120 nm).	*In vivo* (PC-3 tumor xenograft).	Nanobrachytherapy for prostate cancer.	After 5 weeks of radioactive injection (1.5 mCi per mouse), the decrease in tumor volume by about 75% for the ^103^Pd@Au-treated group was reported, and over 95% of NPs still remained in the tumor.	(72)
Auger electrons	AuNPs radiolabeled with ^111^ln (30 nm) using DTPA chelator and functionalized with PEG and trastuzumab.	*In vivo* (subcutaneous HER2-overexpressing breast cancer (BC) xenografts).	HER-2-positive BC cells.	Therapeutic effectiveness of trastuzumab-AuNP-^111^ln was assessed by intratumorally injecting 10 MBq of radiopharmaceutical to the BC murine model. Inhibition in growth of tumor was reported for the treated group, whereas in the case of an untreated group, the tumors grew to eightfold of the initial tumor size.	(52)
Auger electrons	^103^Pd core coated with Au or ^198^Au (5–30 nm) functionalized with PEG.	*In vivo* (PC-3 tumor xenograft).	PC-3 prostate cancer cells.	4 weeks post radioactive injection (single dose of 1.6–1.7 mCi per mouse), a delay in tumor growth by 56% and 75% was reported for ^103^Pd@AuNPs and ^103^Pd@^198^AuNPs, respectively, with respect to the controls. 75% of the injected dose was detected in the tumor.	(15)
Auger electrons	Covalent organic frameworks (COF)-Ag particles conjugated with PEG and radiolabeled with ^125^I.	*In vivo* (PC-3 tumor xenograft).	PC-3 prostate cancer cells.	For the ^125^I-COF-treated group (injected with 1 mCi of PEG-COF-Ag-^125^I per mouse), reduction in tumor volume by about 63% in comparison with the initial size was reported.	(45)
Auger electrons	Nanogel with ^103^Pd-AuNPs coated with poly(*N*-isopropylacrylamide) (37.3 nm).	*In vivo* (CT26 colorectal tumor xenograft).	CT26 colorectal cancers.	The delay in the tumor growth for treated group (injected with 25 MBq of radioactive LOIB : EtOH-[^103^Pd]AuPd nanogel) after day 10 p.i. was reported in comparison with the control and cold nanogel groups. Further, the *ex vivo* biodistribution studies elucidated that up to 95%ID/g of injected radioactive nanogel was retained in the tumor post day 20 of injection.	(38)
β	^198^Au-poly(amidoamine) dendrimer nanoparticles (10–50 nm)	*In vivo* (B16F10 melanoma tumor model).	B16F10 tumor cells.	Reduction in tumor growth by more than 45% was observed for Group B mice (injected with 74 μ of poly{^198^Au}) in comparison with the control and Group A mice (injected with 35 μCi of poly{^198^Au}).	(11)
β	^198^AuNPs stabilized with gum arabic (4–10 nm).	*In vitro* (PC-3 tumor cell lines) and *in vivo* (PC-3 tumor xenograft)	PC-3 prostate cancer cells.	*In vitro* stability studies demonstrated excellent stability of GA-^198^AuNPs for periods of over 6 months. The biodistribution studies performed in a murine model demonstrated that more than 85% of GA-^198^AuNPs were contained in the liver.	(73)
β	^198^AuNPs stabilized with gum arabic (12–18 nm).	*In vivo* (PC-3 tumor xenograft).	PC-3 prostate cancer cells.	After 3 weeks of radioactive injection (408 μCi of GA-^198^AuNP per mouse), the tumor volumes of treated groups were found to be 82% smaller than those of the control group. Furthermore, even after 30 days of injection, on *ex vivo* analysis, radioactive nanoparticles were found in the tumor (20% ID), the liver (1% ID), and the carcass (18.5% ID).	(35)
β	^198^Au-EGCg nanoparticles	*In vivo* (PC-3 tumor xenograft).	Lam 67R receptors in prostate cancer cells.	After 24 h of radioactive injection (136 μCi of ^198^Au-EGCg nanoparticles per mouse), approximately 72% of nanoparticles were retained in the tumor. After 28 days of injection, the tumor size of the treated group was found to be 80% smaller than that of the control group.	(36)
β	^198^AuNPs stabilized with gum arabic (12–15 nm).	*In vivo* (dogs diagnosed with prostate cancer).	Spontaneous prostate cancer in dogs.	The dogs were injected with activity in the range of 3 to 13.8 mCi of ^198^Au. A decrease in tumor volume by 30%–50% was observed in two specimens; an increase in tumor size by 12%–26% was observed in 2 dogs; and for the remaining specimens, there was an increase or decrease of 3% in tumor volume (probably due to limited retention in the tumor volume).	(74)
β	Mangiferin-^198^Au nanoparticles (35 nm)	*In vivo* (PC-3 tumor xenograft).	PC-3 prostate cancer cells.	Mice bearing prostate cancer were divided into three groups: Group A and Group B were injected with 160 μCi/30 μL of MGF-^198^AuNPs, and Group C was injected with 30 μL of saline. After 2 weeks of injection, a decrease in tumor volume by 2 fold with respect to control was reported for the treated groups. Three weeks post radioactive injection, there was an increase in tumor volume by fivefold for Group C; Group A = 0.18 ± 0.17 cm^3^ and Group B = 0.22 ± 0.02 cm^3^ were reported. Furthermore, after 3 weeks, 69.70 ± 14.40%ID was found to be retained in the tumor, 6.80 ± 5.9%ID in the carcass, and 1.44 ± 2.97%ID in the liver.	(10)
β	^198^AuNPs stabilized with gum arabic (~2 nm).	*In vivo* (H460 tumor xenograft).	H460 non-small cell lung cancer cells.	Post 7 days of injection (103 μCi of ^198^AuNPs@GA per mouse), a decrease in tumor volume by more than 90% was observed in the ^198^AuNPs@GA-treated group in comparison with the controls and mice injected with non-radioactive nanoparticles. Even after 2 weeks of radioactive injection, 50% of the nanoparticles were found to be accumulated in the tumor and 8.9% in the liver.	(49)
β	AuNPs radiolabeled with ^177^Lu *via* DOTA chelator, functionalized with PEG and panitumumab.	*In vivo* (MDA-MB-468 human breast cancer mice model)	MDA-MB-468 human breast cancer cells.	A single dose of 4.5 MBq of ^177^Lu-AuNP was intratumorally administered to the mice carrying subcutaneous BC cells. No significant impact of active targeting of ^177^Lu-AuNP was observed in retaining the AuNPs within the tumors. Less than 3%ID/g radioactivity migrated to the liver and spleen, and its value increased by two to fivefold post 48 h of injection, whereas the radioactivity found in other organs was less than 0.5%ID/g. In the treated groups, inhibition of tumor growth by a factor of ≈30 in comparison with the untreated groups was reported.	(50)
β	AuNPs radiolabeled with ^177^Lu *via* DOTA chelator, functionalized with PEG and trastuzumab (30 nm).	*In vivo* (breast cancer xenografts).	BC tumor cells.	3 MBq of ^177^LuAuNPs was injected intratumorally to each mouse. The targeted nanoparticles (trastuzumab-AuNP-^177^Lu) were reported to be 1.8 times more efficient in inhibiting tumor growth in comparison with the non-targeted (AuNP-^177^Lu) and 2.2 times in comparison with the untreated group.	(75)
β	^199^AuNPs stabilized with [f(RGDfK)] peptide (11 nm).	*In vivo* (melanoma tumor xenograft).	Integrin α_v_β_3_ receptors in melanoma cells.	Significant delay in tumor growth was observed in mice injected with 2, 5, or 10 MBq of ^199^Au-c(RGDfK) nanoparticles in comparison with the control.	(37)
β	Melanin-silver nanoparticles radiolabeled with ^131^I cyan (6 nm).	*In vivo* (PC-3 tumor xenograft).	PC-3 prostate cancer cells.	The MNP-Ag-^131^I-treated group (injected with 500 mCi of ^131^I) had tumor volume equal to initial volume, whereas the control and ^131^I-treated group had tumor size 1.5 times larger in comparison with the initial volume.	(17)
β	Mesoporous silica nanoparticles radiolabeled with ^131^I and activated with anti-VEGFR2 antibodies and bovine serum albumin.	*In vivo* (thyroid cancer-bearing mice).	VEGFR2 in human thyroid carcinoma FRO cells.	The mice were intratumorally administered with a single dose of 74 MBq of radioactive nanoparticles. Gradual increase in tumor volume was reported for all the groups except ^131^I-BSA-MSNPs-anti-VEGFR2-treated group.	(76)
β	AuNPs radiolabeled with ^131^I and activated with twin arginine translocation (TAT) peptide (~8.36 nm).	*In vivo* (HCT-116 colon cancer xenografts).	Human colon cancer (HCT-116) cells *in vivo*.	After 18 days of radioactive injection (500 μCi/mL per mouse), reduction in tumor size by 79.95% was reported for the ^131^I-AuNPs-TAT-treated group, whereas in the untreated group, the tumor grew to 8.08 times the original tumor size.	(77)

### 5.1 Alpha Emitters

Dziawer et al. (51) synthesized AuNPs at diameters of 5 and 15 nm. The nanoparticles were functionalized with Substance P(5-11) [SP(5-11)] peptide fragment to actively target the NK1 receptors overexpressed by T98G glioma cells. The AuNP-S-PEG-SP(5-11) bioconjugate was radiolabeled by adsorbing ^211^At on the surface of AuNPs. The *in vitro* cytotoxicity of the obtained ^211^At-AuNP-S-PEG-SP (5-11) radiobioconjugate was evaluated in human serum and cerebrospinal fluid. No study on therapeutic efficacy and *in vivo* biodistribution of radiobioconjugate has been reported. However, the authors recommended the intratumoral injection of ^211^At-AuNP-S-PEG-SP (5-11) radiopharmaceutical, instead of intravenous injection due to its large size.

Recently, the same group synthesized 5-nm-sized AuNPs, with ^211^At chemically adsorbed on its surface for nanobrachytherapy purposes using alpha emitters [56]. The nanoparticles were activated with PEG and trastuzumab (antibody) to actively target HER-2 proteins overexpressed on the surface of ovarian cancer-derived SKOV-3 cells. In the *in vitro* study, the authors demonstrated that AuNP-S-PEG-trastuzumab bioconjugate was effectively internalized by SKOV-3 cells. Furthermore, an *in vitro* cell viability test demonstrated that ^211^At-AuNP-trastuzumab radiobioconjugate effectively reduced the metabolic activity of ovarian cancer cells with a median lethal dose of 0.5 MBq/mL. In this case as well, no biodistribution or therapeutic evaluation was reported.

Salvanou et al. (14) synthesized AuNPs radiolabeled with ^225^Ac *via* DOTA-derivative (TADOTAGA) chelator. The chelator TADOTAGA formed a strong bond with the AuNPs resulting in the formation of a highly stable colloid in aqueous medium, and the chelating characteristics of DOTA-derived macrocyclic compound were exploited to radiolabel the Au@TADOTAGA nanocarriers. The [^225^Ac]^225^Ac-Au@TADOTAGA nanoparticles (5–9 nm) were synthesized with radiochemical yield of 86% and radiochemical purity greater than 93%. The aim of the study was to evaluate [^225^Ac]^225^Ac-Au@TADOTAGA nanoparticles as a nanobrachytherapy agent. The radiolabeled nanoparticles were evaluated in terms of i) its stability and *in vitro* cytotoxicity in U-87 MG (human glioblastoma–astrocytoma) cancer cells and ii) *in vivo* biodistribution by intravenous (i.v.) and intratumoral injection of [^225^Ac]^225^Ac-Au@TADOTAGA nanoparticles to the mice bearing U-87 tumor. Additionally, the tumor regression studies were performed over a period of 22 days to evaluate the therapeutic efficacy of intratumorally injected ^225^Ac radiolabeled nanoparticles. For *in vivo* biodistribution studies, the mice (tumor volume = 200–400 mm^3^) were divided into two groups, with three to five mice in each group. The [^225^Ac]^225^Ac-Au@TADOTAGA (100 μ, ≈1 kBq per mouse) nanoparticles were injected intravenously to the first group and intratumorally to the second group. The mice were euthanized at 2, 4, 24, 72, 120, and 288 h after injection; all the major tissues and organs were removed and weighted; and radioactivity was counted in terms of % injected dose per gram (%ID/g). For the first group (i.v. injection) at 2 h post injection (p.i.), the uptake of radiopharmaceuticals in the kidney ≈28%ID/g decreased to ≈9%ID/g at 120 h p.i., which showed the renal clearance of AuNPs, whereas the uptake in the liver and spleen increased from 9.5%ID/g and 7.2%ID/g at 2 h p.i. to 21.5%ID/g and 13.3%ID/g at 120 h p.i. The maximum uptake in tumors (4%ID/g) occurred at 2 h p.i. and decreased to 1%ID/g at 120 h p.i. On the other hand, for the second group (intratumoral injection), the reported tumor uptake was 60.67%ID at 2 h p.i. and decreased to 5.2%ID/g at 228 h p.i. For therapeutic efficacy evaluation, mice with ≈300 mm^3^ of U-87 MG tumor xenograft were again divided into two groups. The first group (control) was injected intratumorally with 100 μL of saline, and the second group (therapy group) was injected with 100 μL/5 kBq of [^225^Ac]^225^Ac-Au@TADOTAGA on days 1, 3, and 5; and the tumor volume was tracked over 22 days. The tumor volume of therapy group was reported ≈2.4 times lower after 8 days of radioactive injection and ≈4 times lower after 22 days of injection, in comparison with the control group.

### 5.2 Auger Emitters

Moeendarbari et al. (72) reported the synthesis of nanoparticles radiolabeled with ^103^Pd for nanobrachytherapy applications. A monodispersed layer of ^103^Pd was coated on gold spherical shells, hence synthesizing ^103^Pd@Au nanoseeds with a diameter of approximately 120 nm. These nanoseeds were injected intratumorally to mice bearing prostate cancer tumors to evaluate their *in vivo* therapeutic efficacy and biodistribution. The mice were randomized into three groups (n = 6), treated with phosphate-buffered saline (PBS) solution, non-radioactive (cold) Pd@Au nanoparticles in PBS suspension, and radioactive ^103^Pd@Au nanoparticles in PBS suspension. In order to achieve uniform distribution of radiation dose in the whole tumor mass (181.7 ± 62.1 mm^3^), the intratumoral injection was injected at six to nine locations, and radioactivity of 1.5 mCi per tumor was injected. The total injected volume of PBS, cold Pd@AuNPs, and ^103^Pd@AuNPs was kept below 40 μL. The evaluation of retention of nanoseeds within the tumor volume and their migration to other organs was performed *ex vivo* and with single-photon-emission CT (SPECT)/CT. Upon SPECT/CT imaging, it was reported that after day 1 of radioactive injection, 101.50 ± 23.72%ID/g was retained within the tumor volume, and a negligible amount of radioactivity (≈0.1%ID/g) was observed in the liver and spleen. Furthermore, after 5 weeks of radioactive injection, 274.5 ± 77.6%ID/g was detected in the tumor volume, as the tumor volume decreased over the course of the treatment. This indicated the expected radiotherapeutic effect of the ^103^Pd@Au nanoseeds. Furthermore, the *ex vivo* biodistribution investigation (5 weeks p.i.) results showed that ≈95% of nanoseeds were retained within the tumor, ≈3% migrated to the liver, and approximately 0.5% were found in the spleen. In terms of therapeutic efficacy, after 5 weeks of radioactive injection, the decrease in the tumor volume by about 75% for the ^103^Pd@Au treated group was reported, whereas the increase in the tumor volume for groups treated with PBS and cold nanoparticles was reported.

Cai et al. (52) synthesized AuNPs radiolabeled with ^111^ln of 30-nm diameter. The radionuclides (^111^ln) were attached to the AuNPs using DTPA. The nanoparticles were also functionalized with PEG chains linked to antibody trastuzumab. Consequently, trastuzumab-AuNP-^111^ln radiopharmaceutical was obtained. Trastuzumab was used to actively target HER2-positive BC cells. The authors evaluated the *in vitro* cytotoxicity of synthesized radiolabeled nanoparticles on HER2-positive BC cells. Additionally, *in vivo* therapeutic effectiveness of trastuzumab-AuNP-^111^ln was also assessed by intratumorally injecting 10 MBq (≈270 µCi) of radiopharmaceutical into subcutaneous HER2-overexpressing BC xenografts. Tumor growth in the BC murine model was monitored for more than 70 days post radioactive injection. Inhibition in growth of tumor was reported for the treated group, whereas in the case of the untreated group, the tumors grew up to eightfold of the initial size. Tissue toxicity was not observed. No information regarding the migration of radiolabeled AuNPs to the liver and spleen was provided, as the authors did not perform biodistribution evaluation.

Laprise-Pelletier et al. (15) synthesized two types of radio-NPs composed of a nanoscopic core of radioactive palladium (^103^Pd : Pd) coated with gold (Au)-^103^Pd : Pd@Au and ^103^Pd : Pd@^198^Au : Au. These nanoparticles were synthesized using chemical reduction technique, one-pot method. In ^103^Pd-Au nanoparticles, the ^103^Pd : Pd radioactive core served the purpose of low-energy photon source, and the outer gold (Au) shell provided biocompatibility and protection and enhanced the radiation dose delivered by the process of radiosensitization. Additionally, ^103^Pd-Au nanoparticles were labeled with ^198^Au (high-energy beta emitter). In order to minimize the absorption of Auger and delta electrons by gold, the core size was kept at the range of 5–30 nm. The nanoparticles were synthesized with radiochemical yield of 87%. These nanoparticles were further functionalized with PEG; ^103^Pd : Pd@Au-PEG and ^103^Pd : Pd@^198^Au : Au-PEG nanoparticles were synthesized. In order to assess the therapeutic efficacy of both types of nanoparticles, a single dose of 1.6–1.7 mCi (2–4 μL) was intratumorally injected to the mice with prostate cancer tumors (PC-3 cell lines). Four weeks post radioactive injection, a delay in tumor growth by 56% and 75% was reported for ^103^Pd : Pd@Au-PEG NPs and ^103^Pd : Pd@^198^Au : Au-PEG NPs, respectively, with respect to the controls. Through biodistribution evaluation, the authors demonstrated that most of the nanoparticles were retained within the tumor, as more than 75% of the total radioactivity measured in the mice at the time of euthanasia was found there. Additionally, up to 16% of nanoparticles were found in the liver, 3% in the spleen, and less than 1% in other organs.

Zhang et al. (45) used COFs to synthesize nanoparticles radiolabeled with ^125^I. Initially, Ag^+^ ion was attached to the N atom of the bipyridine group present on 2,2′-bipyridine-based COF, and COF-Ag bioconjugate was formed. This bioconjugate was functionalized with PEG and radiolabeled with ^125^I, consequently resulting in the formation of PEG-COF-Ag-^125^I nanoparticles with radiolabeling yield of 94% and stability of more than 90% (after 7 days) in PBS and serum. The authors also evaluated the *in vitro* radiotoxicity of PEG-COF-Ag-^125^I nanoparticles on PC-3 cell lines with variable activity (0–200 Ci/mL). The decrease in the survival of PC-3 cells by 25.8% was reported. Furthermore, the therapeutic efficacy of the ^125^I radiolabeled nanoparticles was also evaluated. To evaluate the therapeutic efficacy, the mice were divided into three groups: i) control, injected with 50 μL of PBS; ii) ^125^I group, injected with 1 mCi of ^125^I in 50 μL of PBS; and iii) ^125^I-COF group, treated with 1 mCi of PEG-COF-Ag-^125^I radiobioconjugate. The radiopharmaceutical and PBS were injected intratumorally to the mice. The activity retention time was studied through SPECT/CT at 0.5, 10, 24, and 36 h p.i. The authors demonstrated that at 0.5 h p.i., signal intensity was 3.2 times higher at tumor site for ^125^I-COF group in comparison with ^125^I group. On average, 61.67% of PEG-COF-Ag-^125^I nanoparticles were retained in the tumor volume. Based on the data of time of retention of nanoparticles in the tumor volume, all three groups were reinjected with PBS, 1 mCi of ^125^I, and 1 mCi of PEG-COF-Ag-^125^I after 4 days; and the mice were euthanized 9 days after the first day of radioactive injection. For the ^125^I-COF group, the reduction in the tumor volume by about 63% in comparison with the initial size was reported. Additionally, an increase in the tumor size by factor of 2 for control and ^125^I group with respect to the initial tumor size was reported. The authors did not perform the biodistribution evaluation of the radio-NPs.

Fach et al. (38) synthesized [^103^Pd]AuPd radio-NPs using chelator-free radiolabeling technique. The [^103^Pd]Pd^2+^ was reduced in the presence of Au^3+^ and citric acid to form [^103^Pd]AuPd radio-NPs of 15-nm size and 23.5-nm hydrodynamic diameter. The radio-NPs were coated with a biocompatible polymer, poly (*N*-isopropylacrylamide) (PNIPAAm), resulting in the formation of hydrophobic [^103^Pd]AuPd radio-NPs of 37.3-nm diameter. The PNIPAAm-coated radio-NPs were further mixed with sucrose acetate isobutyrate (SAIB) or lactose octaisobutyrate (LOIB) in the presence of ethanol. A biocompatible, low-viscosity, injectable LOIB : EtOH radioactive “nanogel” containing [^103^Pd]AuPd was synthesized. The therapeutic efficacy of the radioactive nanogel was assessed on mice with syngeneic CT26 colorectal cancers. The mice were divided into three groups: i) control group: the intratumoral injection was mimicked by inserting a syringe needle into the tumor, and nothing was injected. ii) “Cold nanogel” group: 50 μL of LOIB : OH bioconjugate was injected into the tumor through intratumoral injection. iii) Treated group: 0.675 μCi (25 MBq) was injected into 50 μL of radioactive LOIB : EtOH-[^103^Pd]AuPd nanogel. The delay in the tumor growth after day 10 p.i. was reported in comparison with the control and cold nanogel groups. Further, the *ex vivo* biodistribution studies elucidated that up to 95%ID/g of injected radioactive nanogel was retained in the tumor post day 20 of injection and less than 0.01%ID/g of nanogel was found in the kidney, liver, spleen, and muscles of the mice. Additionally, the authors found no evidence of release of radioactivity from the LOIB : EtOH gel.

### 5.3 Beta Emitters

Khan et al. (11) synthesized radioactive polymerized gold-dendrimer (poly{^198^Au}) nanoparticles using poly(amidoamine) (PAMAM) dendrimers and chloroauric (HAuCl_4_) acid for nanobrachytherapy applications. The steps involved in the synthesis of gold-dendrimer nanoparticles were formation and decomposition of dendrimer-amine[AuCl4] complex, followed by reduction of Au^3+^ to Au. Consequently, positively charged poly{^197^Au} nanoparticles of 10- to 50-nm size range were fabricated. The positive charge of these nanocarriers was expected to enhance the internalization of nanoparticles within the tumor cells. Furthermore, the 10- to 50-nm size range was used to take advantage of enhanced permeability and retention (EPR) effect. EPR effect is increased in accumulation of nanoparticles within the tumor due to the porosity and irregularity in the tumor microvasculature. The aqueous solution of poly{^197^Au} nanoparticles was irradiated with neutron beam, and poly{^198^Au} was obtained. The therapeutic efficacy studies of poly{^198^Au} were performed on C57BL/6J mice having B16F10 melanoma tumor model. At the time of treatment, mice were approximately 8 weeks old and had tumor size of 440 to 530 mm^3^. For therapeutic evaluation, the mice were divided into three groups, with each group having seven mice: i) Group A was administered 35 μCi of poly{^198^Au}, in PBS, intratumorally; ii) Group B received 74 μCi of poly{^198^Au} in PBS through intratumoral injection; and (iii) Group C was injected with 75 μL of PBS per mouse. The tumor size was monitored for 8 days post radioactive injection. Group A mice (treated with 35 μCi of poly{^198^Au}) showed a delay in tumor growth in comparison with the control (Group C). However, the difference was not statistically significant. Reduction in tumor growth by more than 45% was observed for Group B mice (injected with 74 μCi of poly{^198^Au}) in comparison with the control and Group A. The authors did not perform biodistribution studies.

A research group from the University of Missouri used phytochemicals to synthesize radioactive AuNPs through chemical reduction techniques (35, 73). In their first research work, they reported the production of AuNPs using gum arabic (GA) solution. The GA-coated radioactive AuNPs (GA-^198^AuNPs), with a diameter of 4–10 nm, were synthesized by adding tris hydroxymethyl phosphine-aniline (P(CH_2_NHCH(CH_3_)-COOH)_3_ (a reducing agent) and GA to H^198^AuCl4 (73). Here, GA was used as a stabilizing agent. *In vitro* stability studies demonstrated excellent stability of GA-^198^AuNPs for periods of over 6 months. The biodistribution studies performed in a murine model demonstrated that more than 85% of GA-^198^AuNPs were contained in the liver. Additionally, the authors performed detailed *in vivo* therapeutic assessments, where GA-^198^AuNPs (diameter 12–18 nm) were injected intratumorally to the severely compromised immunodeficient (SCID) mice bearing prostate tumor (PC3 cells) xenografts. Each mouse was given an intratumoral injection of 408 μCi of GA-^198^AuNPs (30 μL). The tumor volume was monitored over a period of 30 days, and retardation in tumor growth for the treated group in comparison with the untreated group was reported. After 3 weeks of radioactive injection, the tumor volumes of treated groups were found to be 82% smaller than in the control group. Furthermore, even after 30 days of injection, on *ex vivo* analysis, radio-NPs were found in the tumor (20%ID), the liver (1%ID), and the carcass (18.5%ID) (78). In recent years, the researchers from the University of Missouri have developed similar products and tested the radio-NPs *in vivo* as potential nanobrachytherapeutic agents.

Shukla et al. (36) synthesized radioactive AuNPs functionalized with epigallocatechin gallate (EGCg)-^198^Au-EGCg. EGCg is a phytochemical extracted from green tea leaves and can be used to actively target laminin receptors (Lam 67R), which are overexpressed by the prostate cancer cells. In this study, i) the synthesis and characterization of ^198^Au-EGCg nanoparticles were reported; ii) the affinity of EGCg for laminin receptors and internalization of ^198^Au-EGCg through endocytosis was demonstrated; (iii) *in vivo* therapeutic assessment of ^198^Au-EGCg nanoparticles was performed. For *in vivo* therapeutic assessment, 136 μCi (30 μL) of ^198^Au-EGCg nanoparticles, with a diameter of 40–55 nm, were injected intratumorally to the mice bearing prostate tumor. The pharmacokinetic study results demonstrated that after 24 h of injection, approximately 72% of ^198^Au-EGCg nanoparticles were retained in the tumor. After 28 days of injection, the tumor size of the treated group was found to be 80% smaller than of the control group. The results of end-of-study biodistribution, conducted on day 42 post radioactive injection, showed that radio-NPs were retained in the tumor (34.7%ID), liver (2.5%ID), and carcass (18%ID).

The therapeutic effectiveness of GA-coated AuNPs (GA-^198^AuNPs) was also assessed in the canine model (74). Nine dogs diagnosed with prostate cancer were injected with GA-^198^AuNPs (diameter 12–15 nm) intratumorally. In order to obtain homogeneous distribution of a radiotherapeutic agent within the tumor volume, two to eight needles were inserted, and several injections of 100–200 μL were administered. Activity to be administered was selected as a function of tumor volume. The dogs were injected with activity in the range of 3 to 13.8 mCi of ^198^Au. This activity range corresponded to the biological effective dose of 50 (n = 2) and 150 Gy (n = 7). After 30 min of radioactive injection, scintigraphy scans were performed. In six dogs, the migration of nanoparticles to the bladder, urethra, and prostatic extra region from the prostate was observed. After 30 min of injection, only 53% of injected radio-NPs were retained in the prostate. Four weeks posttreatment, CT scan was performed to measure the tumor volume. The authors expressed the effectiveness of the treatment in terms of decrease in the tumor volume. A decrease in the tumor volume by 30%–50% was observed in two specimens, an increase in tumor size by 12%–26% was observed in two dogs, and for the remaining specimens, there was an increase or decrease of 3% in the tumor volume. The nanoparticles did not induce any sign of toxicity. The authors concluded that the therapeutic effectiveness of GA-^198^AuNPs in the canine model was compromised due to the limited retention of radio-NPs within the tumor volume. Hence, the influence of tumor vasculature and the lymphatic drainage on retention or leakage of nanoparticles need to be investigated before conducting clinical trials.

In the most recent publication from this group (10), they used mangiferin (MGF), a phytochemical extracted from mango, to fabricate ^198^Au nanoparticles. Mangiferin is a glucose-functionalized xanthonoid and is capable of reducing ^198^Au precursors to ^198^Au nanoparticles. The sugar-polyphenolic groups present in mangiferin are capable of encapsulating and binding on the surface of AuNPs and provide optimum stability both *in vitro* and *in vivo*. Hence, MGF-encapsulated ^198^AuNP-MGF-^198^AuNPs with 35 ± 2 nm of core size and 55 ± 0.9 nm of hydrodynamics size were fabricated. Furthermore, due to the presence of glucose functionality, MGF was used to effectively target laminin receptors overexpressed by the prostate cancer (PC-3) tumor cells. Hence, selective accumulation of MGF-^198^AuNPs within the tumor volume was achieved. The authors reported the following: i) the fabrication and characterization of MGF-^198^AuNPs; (ii) studies on stability of MGF-^198^AuNPs *in vitro* and *in vivo* and biodistribution studies; and (iii) studies on the evaluation of therapeutic efficacy of MGF-^198^AuNPs on mice bearing prostate tumors. In order to evaluate the *in vivo* stability, normal mice (N = 25) were given intravenous injection of 8.0 μCi/100 μL of MGF-^198^AuNPs and were euthanized at 30 min, 1 h, 2 h, 4 h, and 24 h post radioactive injection. All the important organs (liver, spleen, lungs, bladder, etc.) were collected, and radioactivity accumulation in these organs was estimated. MGF-^198^AuNPs predominantly accumulated in the spleen and liver clearance through hepatobiliary pathway, and almost no uptake occurred in the blood and lungs. In order to evaluate the selective accumulation of MGF-^198^AuNPs, due to the glucose functionality of MGF, the authors performed a study on the retention of radiopharmaceuticals within the tumor. Mice bearing PC-3 tumor (N = 5) were administered with a single dose of 4 μCi/30 μL of MGF-^198^AuNPs for each tumor through intratumoral injection. The mice were euthanized at an interval of 30 min, 1 h, 2 h, 4 h, and 24 h post radioactive injection; and tumors and the organs of interest (liver, spleen, etc.) were excised. Radioactivity accumulation in tumor and different organs was estimated in terms of %ID/organ. At 30 min and 24 h p.i., 80.98% ± 13.39% and 79.82% ± 10.55% of MGF-^198^AuNPs were respectively found to be accumulated in the tumor, whereas liver increase from 4.05% ± 5.27% (at 30 min) to 10.65% ± 8.31% (at 24 h) was reported. Additionally, low uptake of radio-NPs was also found in feces (0% at 30 min and 2.2% ± 4.5% at 24 h) and the stomach (0.10% at 30 min and 0.02% at 24 h), and no noticeable uptake was found in the lungs, blood, and other organs. Lastly, the authors also performed a detailed study to evaluate the therapeutic efficacy of MGF-^198^AuNPs. Mice bearing PC-3 tumors were divided into three groups: i) Group A, tumor volume ranging from 0.15 to 0.2 cm^3^; ii) Group B, mice with tumor volume about 0.43 cm^3^ were injected with a single dose of 160 μCi/30 μL of MGF-^198^AuNPs per tumor through intratumoral injection; and iii) Group C, mice with 0.15 to 0.2 cm^3^ of tumor size were injected with 30 μL of saline intratumorally and served as control. The tumor volume was monitored for 3 weeks. Post 7 days of injection, a decrease in the tumor volume for Groups A and B was observed. After 2 weeks of injection, a decrease in the tumor volume by twofold with respect to control was reported for the treated groups. Three weeks post radioactive injection, there was an increase in the tumor volume by fivefold for Group C; Group A = 0.18 ± 0.17 cm^3^ and Group B = 0.22 ± 0.02 were reported. Furthermore, after 3 weeks, 69.70 ± 14.40%ID was found to be retained in the tumor, 6.80 ± 5.9%ID in the carcass, and 1.44 ± 2.97%ID in the liver.

Lin et al. (49) fabricated AuNPs stabilized with GA-AuNPs@GA of ≈2-nm size for nanobrachytherapy applications. The X-ray irradiation of HAuCl_4_ and GA resulted in the formation of AuNPs@GA. AuNPs@GA nanocarriers were made radioactive through neutron activation, and ^198^AuNPs@GA were obtained. Radiotherapeutic efficacy, biodistribution, and toxicity studies were performed on mice bearing H460 tumor. Suspension of 103 μCi (injection volume = 100 μL) of ^198^AuNPs@GA nanoparticles per mouse was administered intratumorally to the mice bearing H460 tumors, and the tumor volume was monitored for 2 weeks. Toxicity caused by administration of ^198^AuNPs@GA nanocarriers was evaluated in terms of loss in body weight. Less than 20% decrease in body weight was found post 4 days of radioactive injection; and post 7 days of injection, the body weight was recovered. Hence, the authors effectively showed that ^198^AuNPs@GA are safe for treatment. In order to perform biodistribution studies, mice were euthanized, and important organs (liver, spleen, kidney, carcass, etc.) were collected. The authors also collected urine and feces. Post 7 days of injection, a decrease in the tumor volume by more than 90% was observed in the ^198^AuNPs@GA-treated group in comparison with the controls and mice injected with non-radioactive nanoparticles. Even after 2 weeks of radioactive injection, 50% of the nanoparticles were found to be accumulated in the tumor and 8.9% in the liver. Furthermore, clearance of ^198^AuNPs@GA was observed in urine and feces.

Yook et al. (50) evaluated the therapeutic efficacy of radioactive AuNPs in a MDA-MB-468 human BC model. The AuNPs were radiolabeled with ^177^Lu using a macrocyclic complex: 1,4,7,10-Tetraazacyclododecane-1,4,7,10-tetraacetic acid (DOTA) and NPs)were functionalized with PEG and panitumumab (an antibody) to target the epidermal growth factor receptors (EGFRs). The EGFRs are overexpressed by the BC tumor cells. Radio-NPs were divided into two categories: i) targeted—functionalized with PEG and panitumumab-^177^Lu-T-AuNP; and ii) non-targeted—functionalized with PEG but not panitumumab-^177^Lu-NT-AuNP. A single dose of 4.5 MBq of both targeted and non-targeted nanoparticles in 30 μL of saline was administered through intratumoral injection into the mice carrying subcutaneous human BC cells. Both targeted and non-targeted ^177^Lu radiolabeled AuNPs were found to be capable of delaying tumor growth for more than 90 days, and no organ toxicity caused by these nanoparticles was reported. In the treated groups, inhibition of tumor growth by a factor of ≈30 in comparison with the untreated groups was reported. The amount of nanoparticles that was retained within the tumor was evaluated by performing SPECT/CT imaging at 1 and 48 h post radioactive injection. *Ex vivo* analysis was also done to assess the distribution of ^177^Lu-T-AuNP and ^177^Lu-NT-AuNP in different organs. Post 1 h of injection, most of the radio-NPs were confined within the tumors, and migration of this radioactive out of tumors was observed at 48 h. Furthermore, the authors reported that high concentrations of both targeted and non-targeted nanoparticles, >300%–400%ID/g, accumulated within the tumors after 1 h of intratumoral administration. Hence, no significant impact of active targeting of ^177^Lu-AuNP was observed in retaining the AuNPs within the tumors. Less than 3%ID/g radioactivity migrated to the liver and spleen, and its value increased by two- to fivefold post 48 h of injection, whereas the radioactivity found in other organs was less than 0.5%ID/g.

Cai et al. (75) radiolabeled AuNPs with ^177^Lu-DOTA to synthesize ^177^Lu-AuNPs. These nanoparticles were further functionalized with trastuzumab antibodies using PEG. Initially, the PEG chains were linked on the AuNPs, and the trastuzumab molecules were attached on these chains. The nanoparticles were categorized into two groups: i) targeted—nanoparticles functionalized with trastuzumab (trastuzumab-AuNP-^177^Lu); and ii) non-targeted—nanoparticles not functionalized with trastuzumab (AuNP-^177^Lu). In order to assess therapeutic effectiveness of these nanoparticles, 3 MBq (≈81 μCi) was administered intratumorally in mice bearing BC tumors. The tumor growth was monitored for 16 days. The targeted nanoparticles (trastuzumab-AuNP-^177^Lu) were reported to be 1.8 times more efficient in inhibiting tumor growth in comparison with the non-targeted nanoparticles (AuNP-^177^Lu) and 2.2 times in comparison with the untreated group. No significant tissue toxicity was reported by the authors for both targeted and non-targeted treatments. Additionally, the authors provided no information on the amount of nanoparticles that migrated to the liver and spleen.

Chakravarty et al. (37) synthesized neutron-activated ^199^Au radio-NPs with an average particle size 11 nm and hydrodynamic size of about 30.2 nm. Cyclic (arginine-glycine-aspartate-phenylalanine-lysine) [f(RGDfK)] peptide was used as both a stabilizing agent and a reducing agent for the synthesis of ^199^Au-c(RGDfK) nanoparticles to target integrin α_v_β_3_ receptors for nanobrachytherapy applications. Additionally, non-targeted ^199^Au nanoparticles were also synthesized by labeling ^199^Au nanoparticles with scrambled sequence of RGD cyclic (arginine-glycine-lysine-phenylalanine aspartic acid [c(RGKfD)]. The non-targeted ^199^Au-c(RGKfD) nanoparticles were used as control. The authors characterized the nanoparticles using numerous analytical techniques to evaluate the particle identity, size, *in vitro* stability, compatibility to biological medium, and suitability for clinical use. The biodistribution studies were conducted in C57BL/6 mice bearing melanoma tumors after intratumoral administration of ^199^Au-c(RGDfK) nanoparticles. The non-targeted ^199^Au-c(RGKfD) nanoparticles were also injected intratumorally to another group of C57L/6 mice having melanoma tumors and were used as control. The mice were euthanized at 24, 72, and 192 h post radioactive injection, and samples of normal tissues and tumor were collected. At 24 h p.i., a high percentage of administered radioactive ^199^Au nanoparticles (both targeted and non-targeted) were retained within the tumor volume. The uptake of targeted ^199^Au-c(RGDfK) nanoparticles (497 ± 56%ID/g) was reported to be higher than that of non-targeted (400 ± 67%ID/g). Between 24 and 192 h post intratumoral injection, a gradual decrease in radioactivity, accumulated in the tumor, was observed for both targeted and non-targeted ^199^Au nanoparticles. Additionally, at 192 h p.i., twofold higher retention of the targeted ^199^Au-c(RGDfK) nanoparticles (375 ± 78%ID/g) in comparison with non-targeted ^199^Au-c(RGDfK) nanoparticles (182 ± 23%ID/g) was observed. Consequently, higher radioactivity was found in the blood for non-targeted nanoparticles in comparison with the targeted nanoparticles, indicating their leakage from the tumor. Post 120 h of injection, the uptake of targeted ^199^Au-c(RGDfK) (≈4%ID/g) in the liver and kidney was found to be three times lower than of non-targeted ^199^Au-c(RGKfD) (≈12%ID/g) nanoparticles. The uptake in the spleen (≈2%ID/g) was nearly equal for both targeted and non-targeted ^199^Au nanoparticles. The uptake of radio-NPs in the remaining organs was less than 1%ID/g. The therapeutic efficacy of these targeted and non-targeted ^199^Au nanoparticles was evaluated on melanoma-bearing C57BL/6 mice. The mice with tumor size approximately 150 mm^3^ were divided into five sets (five mice per set). Each group was given a single intratumoral injection of saline, non-radioactive Au-c(RGDfK), 2 MBq of ^199^Au-c(RGDfK), 5 MBq of ^199^Au-c(RGDfK) nanoparticles, or 10 MBq of ^199^Au-c(RGDfK) nanoparticles. The first two groups were used as control. Furthermore, the tumor volume and body weight of the mice were monitored for 15 days. A significant delay in tumor growth was observed in mice injected with 2, 5, or 10 MBq of ^199^Au-c(RGDfK) nanoparticles in comparison with the control.

Sheng et al. (17) synthesized melanin nanoparticles (MNPs) radiolabeled with ^131^I. The MNPs were radiolabeled with Ag-I two-step method. First, Ag^+^ ions were chelated by MNPs, and ^131^I ions were attached to Ag^+^ ions to form MNP-Ag-^131^I nanoparticles (diameter = 6 nm, and hydrodynamic diameter = 11 nm) with 99% radiolabeling yield. The authors further evaluated the solubility and/or stability of MNP-Ag-^131^I in demineralized water (DI water), PBS, and serum. Additionally, the *in vitro* biocompatibility was tested in PC-3 prostate cancer cells, and no cytotoxicity was observed. In order to evaluate the *in vivo* therapeutic efficacy of MNP-Ag-^131^I nanoparticles, the mice were divided into three groups: i) control, ii) ^131^I group, and iii) MNP-Ag-^131^I-treated group. On day 1, the ^131^I group and MNP-Ag-^131^I-treated group were injected with 1 mCi of ^131^I and MNP-Ag-^131^I through intratumoral injection; and radiopharmaceutical retention within the tumor was observed through SPECT and Cherenkov radiation. On day 3, through intratumoral injections, control, ^131^I group, and MNP-Ag-^131^I-treated group were injected with 20 mL of PBS, 500 mCi of ^131^I in 20 mL of DI, and 500 mCi of MNP-Ag-^131^I in 20 mL of PBS. The mice were euthanized after 7 days of radioactive injection, and tumor and other important organs were collected. The MNP-Ag-^131^I-treated group had a tumor volume equal to the initial volume, whereas the control and ^131^I-treated group had tumor size 1.5 times larger in comparison with the initial volume.

Zhang et al. (76) synthesized mesoporous silica nanoparticles (MSNPs), radiolabeled with ^131^I and activated with anti-vascular endothelial growth factor receptor 2 (anti-VEGFR2) antibodies and bovine serum albumin (BSA) for the treatment of anaplastic thyroid cancers (APCs). The radiolabeling of the MSNPs was performed using Chloramine-T method, resulting in the formation of ^131^I-BSA-MSNPs-anti-VEGFR2 radioactive nanocarriers. *In vitro* cellular uptake of ^131^I-BSA-MSNPs-anti-VEGFR2 in human thyroid carcinoma FRO cell lines was evaluated through confocal imaging, and time-dependent cellular uptake was evaluated by measuring radioactivity using gamma counter. The therapeutic efficacy of radioactive ^131^I-BSA-MSNPs-anti-VEGFR2 was tested on mice bearing FRO tumor cells. The radiopharmaceutical retention within the tumor was measured through SPECT/CT imaging. The mice were divided into four groups: control, injected with PBS; and ^131^I-BSA-MSNPs, Na^131^I, and ^131^I-BSA-MSNPs-anti-VEGFR2-treated groups (n = 3). Each group was administered with radioactivity of 74 MBq (50 μL) through intratumoral injection. A gradual increase in the tumor volume was reported for all the groups except ^131^I-BSA-MSNPs-anti-VEGFR2-treated group.

Su et al. (77) synthesized AuNPs radiolabeled with ^131^I and activated with twin arginine translocation (TAT) peptide. In order to construct ^131^I-AuNPs-TAT radiopharmaceuticals, first, AuNPs (diameter = ≈8.36 nm) were prepared. Later, AuNPs were functionalized with amino-poly(ethylene glycol)-thiol (HS-PEG2000-NH2) to prepare AuNPs-PEG, and they were conjugated with TAT peptide to prepare AuNPs-TAT. Lastly, through iodogen-catalyzed procedure, AuNPs-TAT was radiolabeled with ^131^I to synthesize ^131^I-AuNPsTAT radiopharmaceutical with radiolabeling yield of 96.5% and radiochemical purity above 78%. *In vitro* experiments on radiocytotoxicity, estimating the rate of apoptosis and suppression of tumor cell proliferation, were performed using cell counting kit-8 (CCK-8) assay by exposing human colon cancer (HCT-116) cells to ^131^I-AuNPs-TAT radiopharmaceutical. The authors concluded that, after the addition of TAT peptide and AuNPs, ^131^I-AuNPs-TAT was internalized by the cell nuclei and caused short-term and long-term damage to the tumor cells. From the results of *in vitro* studies, the authors concluded that 500 μCi/mL of ^131^I-AuNPs-TAT (composed of AuNPs = 100 μg/mL and TAT = 10 g/mL) is appropriate for therapeutic studies. Mice bearing HCT166 tumors were used to evaluate the therapeutic efficacy of ^131^I-AuNPs-TAT. Prior to treatment, SPECT/CT imaging was used to monitor the metabolic distribution of intratumorally administered ^131^I-AuNPs-TAT. The authors reported that about 20.09% of ^131^I-AuNPs-TAT was retained at the site of injection after 36 h. Post SPECT/CT imaging, mice were administered with 500 μCi/mL (per mouse) of ^131^I-AuNPs-TAT through intratumoral injection. After 18 days of radioactive injection, reduction in tumor size by 79.95% was reported for the ^131^I-AuNPs-TAT-treated group, whereas in the untreated group, the tumor grew to 8.08 times the original tumor size. The authors concluded that the presence of TAT and AuNPs i) internalized the radiopharmaceutical to the nuclei of the tumor cells, which elevated the DNA damage; and ii) the high-energy beta particles emitted from ^131^I on interaction with Au produced low-energy X-rays—this further reduced the cold spots and induced a strong immune response.

From the data collected regarding alpha emitters in preclinical studies, it is possible to observe that the most commonly used radioisotope is ^211^At followed by ^225^Ac, mostly radiolabeling AuNPs with small diameter (from 5 to 15 nm). The most interesting finding among the collected studies was the fact that intratumoral injection likely leads to a better outcome in terms of cancer ablation compared with intravenous injection. When it comes to Auger emitters, palladium comes into the scene more often, either cold palladium combined with gold in the core of nanoparticles or ^103^Pd as a radionuclide. Other Auger emitters that are also used are ^111^ln and ^125^I, and the intratumoral injection was the chosen route for administering the nanoparticles in all studies recruited for this paper. Interestingly, the tumor ablation appears to be higher than alpha emitters, with tumors decreasing in size from 56% to 75% among the recruited studies. Finally, beta emitters are likely the most effective in ablating solid tumors, with tumor size decreasing more than 80% in various preclinical studies. The most commonly used radionuclides are ^198^Au and ^199^Au followed by ^131^I and ^177^Lu. Again, the administration route for the nanoparticles was the intratumoral injection.

**Figure 4:**
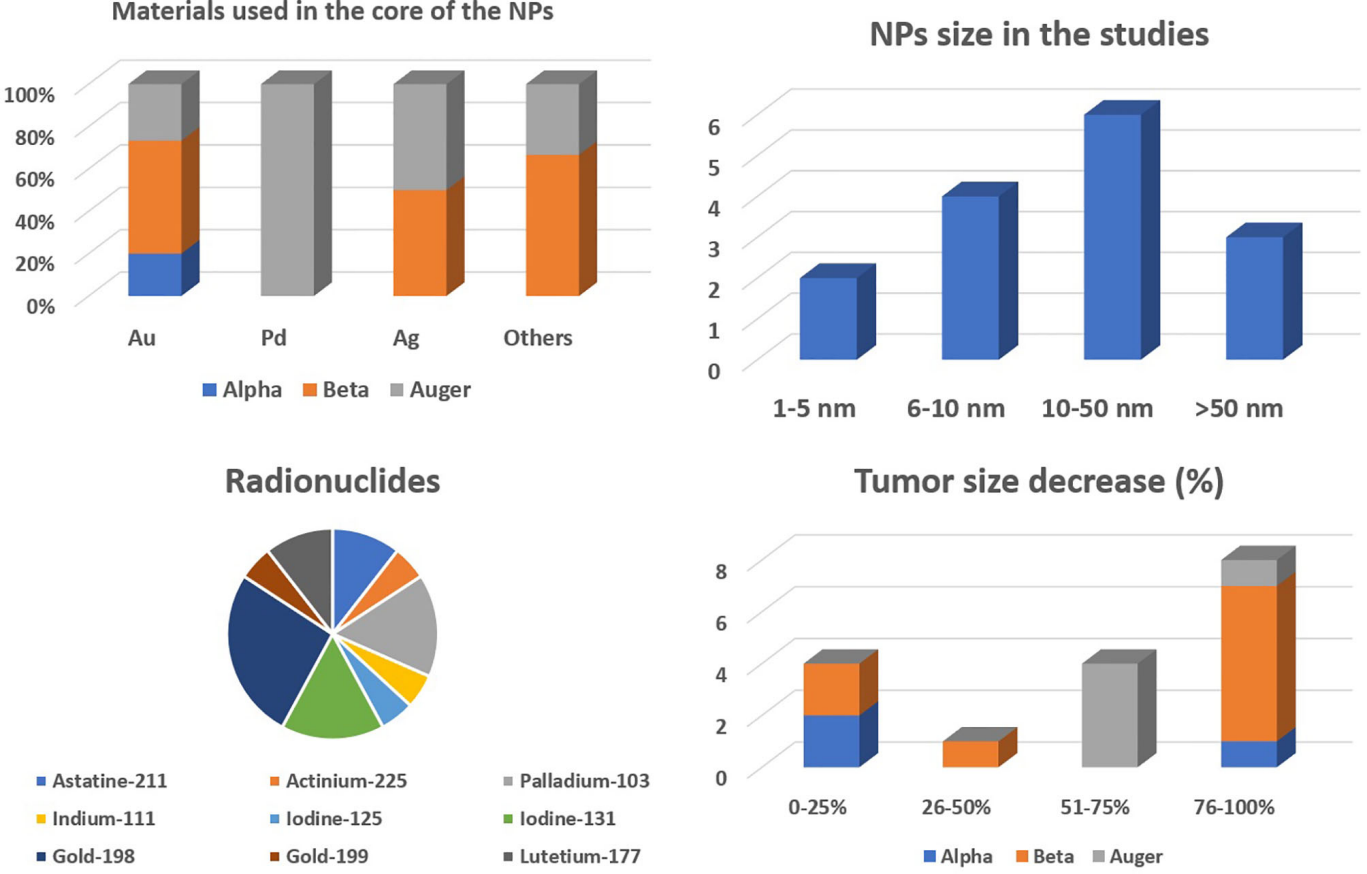
Summary of the main findings from the preclinical studies.

Among the studies recruited for this paper, most of the authors synthesize the nanomaterials, making use of some sort of targeting strategy in order to enhance the tumor localization of the nanoparticles, apart from the intratumoral injection, which also contributes in this regard. However, targeting strategies are more utilized when the authors use beta emitters. Biodistribution studies were performed more often with beta emitters than alpha- or Auger-emitting radionuclides. In this matter, it is of utmost importance to carefully follow the pharmacokinetics of radioactive nanomaterials in order to avoid side effects and non-specific radioactive damage to healthy cells; therefore, researchers should work with novel strategies related to theranostic radioactive nanomaterials. Targeting strategies, i.e., mAb and tissue-specific receptor ligands, are very useful for concentrating therapeutic agents inside the tumor tissues; thus, they should be taken into consideration by those working with this kind of biomaterials. **Figure 4** summarizes the main findings from the preclinical studies.

## 6 Nanobrachytherapy With Intratumoral Immunotherapy

Cancer treatment is a multipronged approach wherein the combination of treatment regimens such as surgery, chemotherapy, RT, and more recently immunotherapy is adopted to achieve better therapeutic index. For example, immunotherapy alone has occasional responses, and benefits are limited to a minority of patients in limited disease sites due to immune evasion properties of tumor cells (79). A rare phenomenon called “abscopal effect” is observed with the local radiation treatments where tumors outside the treatment fields have been observed to shrink as a result of immune response provoked by RT (80–82). The abscopal effect is rare with radiation alone but profoundly observed in patients undergoing immune checkpoint blockade therapy (83–86). Similarly, immunotherapy can enhance the efficacy of RT *via* activation of the innate and adaptive immune system (79, 87). With the development of tumor-specific antibodies, immune checkpoint inhibitor antibodies, and chimeric antigen receptor T-cell therapies, immunotherapy has revolutionized the treatment of metastatic disease including melanoma, non-small cell lung cancer, and renal cell carcinoma (88, 89). The unique synergistic relationship between radiation and immunotherapy provides the benefit of controlling systemic disease with local delivery of treatment. Many clinical trials are ongoing, testing the outcomes (safety, tumor response, immune response, and toxicities) of the combination of immune checkpoint inhibitor therapeutics and RT for prostate cancer (castrate resistant), soft tissue sarcoma, BC, glioma, pancreatic cancer, and melanoma (79, 88, 89). The RT techniques employed are mostly EBRT, including highly conformal intensity modulated RT and stereotactic body RT (SBRT) combined with high-precision image guidance (IGRT). In many studies, immunotherapy was administered intratumorally to envisage lesser immune-related adverse events, better local immune response, and control of systemic metastatic disease (90–96).

In one of the studies, Moreau et al. investigated the use of multifunctional smart RT biomaterial (SRB) loaded with immunoadjuvants to study the abscopal effect of local RT (93). Lewis lung carcinoma (LLC) tumors were generated on both the right and left flanks of the mice, and one tumor was implanted with an SRB device loaded with CD40 antibody in PLGA matrix followed by RT. The SRB device releases immunotherapeutic drug intratumorally and provides image guidance for EBRT using small animal radiation research platform (SARRP). The treatment response was observed in both irradiated and un-irradiated tumors owing to the radiation-mediated systemic antitumor immune response.

There was one attempt to administer both radiation and an immune stimulator directly to the tumor. Sodium alginate formulation containing catalase was labeled with ^131^I and injected intratumorally. This creates *in situ* gelation to confine ^131^I within the tumor and alleviates tumor hypoxia (97). They also showed that when ^131^I was added with CpG oligonucleotide (immunostimulator) administered intratumorally and combined with systemic checkpoint blockade therapy (CTLA-4 antibodies), it leads to local tumor eradication as well as increased systemic immune response to inhibit distal metastasis and tumor recurrence.

Radiation in the form of nanoparticles is being investigated for intratumoral administration to reduce the side effects to normal tissues, and similarly, immunotherapeutic drugs are being administered intratumorally to avoid immune-related adverse events. The therapeutic combination of nanobrachytherapy and intratumoral immunotherapy has great potential to achieve higher therapeutic index in a synergistic manner. They may deliver larger doses of therapeutics to the tumor, reduce normal tissue toxicities of systemic delivery, eradicate distal malignant cells owing to the enhanced abscopal effect, and enhance the efficacy of immunotherapy as well as RT for multiple disease sites and larger patient base.

## 7 Dosimetric Studies on Nanobrachytherapy Applications Using Monte Carlo Methods

Radio-NPs are emerging as promising radiotherapeutic agents for cancer treatment and are being probed as a replacement to seed-based BT. Prior to using radio-NPs for RT applications, accurate dosimetric simulations are needed in order to determine the dose distribution within the tumor volume and the surrounding normal tissues. Monte Carlo simulation techniques can be used efficiently to determine the energy or dose distribution within the region of interest. As per our knowledge, only three studies addressing the problem of dosimetry for nanobrachytherapy using radio-NPs have been published so far. The main highlights of these three studies are briefly discussed below.

Laprise-Pelletier et al. (98) used both experimental and theoretical approaches to estimate the dose distribution maps in the tumor tissues. Initially, radio-NPs ^103^Pd : Pd@Au were synthesized (15) and administered intratumorally to the mice bearing prostate cancer tumors. At different time points (2 h, 24 h, and 8 days), tumors were harvested and analyzed through optical and transmission electron microscopy (TEM). A comprehensive biodistribution study confirmed that more than 80% of radio-NPs were retained in the tumor volume and that a small percentage of NPs migrated to the liver and spleen. The intracellular distribution of ^103^Pd : Pd@AuNPs was quantified through optical and TEM images. Maps and profiles of energy deposition at microscopic and macroscopic levels were estimated using these data. At the macroscopic level, the dose distribution, in terms of isodose curves, obtained for ^103^Pd : Pd@AuNPs (also termed as “cloud” of radio-NPs) was compared with the dose distribution obtained for the conventional millimeter-sized, low-dose BT: ^103^Pd seed. A sharper dose fall in the isodose curves estimated for a single injection of radio-NPs was reported in comparison with the conventional BT seed. This sharper dose fall was attributed to the attenuation of photons by the gold atoms present in the “cloud” of NPs. The authors stressed that this feature can be useful in effectively sparing organs at risk and delivering high doses of radiation to the tumor tissues, as NPs deposit very high doses of radiation in their immediate vicinity. The TEM images of the xenograft tumor cells were used to simulate the energy deposited by ^103^Pd : Pd@AuNPs at the microscopic level. The Monte Carlo simulation was conducted in three steps: i) the TEM images representative of microdistribution of NPs were selected and digitized. These digitized images were virtually placed in the middle of 50-μm^3^ cubical phantom. ii) Nanoconstructs (r = 25 nm) with Pd core (r = 5 nm) coated with a thick layer of gold (r = 20 nm) were simulated. These nanoconstructs were positioned in the resampled TEM images, placed in the cubical phantom. iii) Emitted photons and electrons produced in the interactions were simulated, and energy deposition maps were computed. From the computed dose distribution, it was found that the highest dose deposition occurred in the immediate vicinity of the NPs. That is, the electrons escaping from the NPs lost most of their energy in a very short range, and almost no NPs were found in the nuclei. The same microdosimetric approach was used to quantify the radiosensitization effect induced by gold. In this case, two simulations were performed, considering i) ^103^Pd : Pd core coated with gold and ii) ^103^Pd : Pd not coated with gold. The estimated energy deposition map was reported as a ratio of energy-deposited values (energy deposited by radio-NPs coated with gold/energy deposited by radio-NPs not coated with gold). The ratio of energy-deposited value quantified the dose enhancement effect due to the presence of gold. Enhancement in dose by factor of 25, in the immediate vicinity of the NPs coated with gold, was observed, whereas for regions 2 μm further from the radio-NPs, no radiosensitization effect was observed. Hence, the radiosensitization effect was found to be extremely localized around the gold-coated radio-NPs. Based on the microdosimetric results, the authors concluded that reactive oxygen species (ROS) can be the main factor responsible for cell killing and observed strong tumor control effect (15).

Al-Yasiri et al. (99) used MCNP6.1.1 Monte Carlo (MC) code to construct a simple geometrical replica of a human prostate, containing a tumor inside it, and estimated the dose distribution due to the gold radio-NPs (^198^AuNPs or ^199^AuNPs) homogeneously distributed within the tumor. This simple model consisted of spheres representing the following: tumor (radius (r) = 0.4 cm) located within the prostate (r = 2 cm), prostate, bladder (r = 3.5 cm), and rectum (r = 1.5 cm). Dose distribution was estimated for tumor and other organs at risk (healthy prostate, bladder, and rectum), assuming that 10 mCi of ^198^AuNPs/^199^AuNPs was homogeneously distributed within the tumor volume. The authors reported that for both ^198^AuNPs and ^199^AuNPs, the maximum dose was deposited at the center of the tumor and decreased rapidly towards the tumor prostate interface and other surrounding organs. Owing to the high-energy beta emissions from ^198^Au, high dose rates were reported for ^198^Au at a) center of the tumor, 12 Gy/h; b) prostate tumor periphery, 1.46 Gy/h; c) prostate periphery, 0.1 Gy/h; d) center of the bladder, 0.013 Gy; and e) center of the rectum, 0.026 Gy/h. On the other hand, due to low-energy beta emissions for ^199^Au, for the same locations, the dose rates were 1.6, 0.53, 0.26, 0.0013, and 0.004 Gy/h. Based on these findings, the authors concluded that ^198^AuNPs are suitable for the treatment of solid tumors and that ^199^Au can be used for imaging purposes.

In one of our recent *in silico* dosimetry study (61), we replicated the cell survival curves for three preclinical studies (10, 15, 100), published in literature, on the use of radioactive nanocarriers as nanobrachytherapeutic agents using a mathematical model (101) and EGSnrc (102) MC code. The mathematical model used took into account the doubling rate of tumor cells, complete repair of sublethal damage, uptake rate, and washout rate of nanocarriers to and from tumor cell monoexponential function of time. Furthermore, this study anticipated several possibilities and evaluated the dosimetric characteristics and therapeutic efficacy of nanoparticles radiolabeled with ^103^Pd (Auger emitter), ^153^Sm (medium energy beta emitter), and ^198^Au (high-energy beta emitter). Initially, the dosimetric characteristics of ^103^Pd, ^153^Sm, and ^198^Au were evaluated using single cell dosimetry (7). It was found that at the cellular level, ^153^Sm deposited maximum dose, followed by ^103^Pd and ^198^Au. The least energy deposition for ^198^Au was attributed to the emitted highly energetic beta particles. These beta particles exit the cell volume (radius = 5 μm) without depositing enough energy. Second, the estimated cell survival curves were found to be in good agreement with the experimental results published in literature. Lastly, we evaluated the impact of i) tumor size, ii) tumor type, and iii) amount of injected activity on the cell survival curves. We found that ^153^Sm and ^198^Au effectively ablated tumor cells for all three cases with minimum injected activity (≤20 MBq), whereas for ^103^Pd, higher radioactivity was required to achieve a similar effect. Hence, we concluded that for radioresistant, large size (≈1 cm^3^) and rapidly growing tumors, ^153^Sm and ^198^Au can be conclusively used as nanobrachytherapeutic agents, whereas ^103^Pd is only suitable for small-size (≈0.3 cm^3^) tumors that have injected activity ≥60 MBq.

## 8 Conclusion, Current Challenges, and Future Prospects

The application of interstitial BT is impeded by several posttreatment adverse effects or symptoms and the associated operational and logistical complications. The emerging nano-platforms can be used to efficiently deliver radiopharmaceuticals to the tumor. In comparison with free radioisotopes or radioisotopes functionalized with single tumor-specific biomolecules, nanoparticles can be loaded with higher doses of radioactivity, and multiple radioisotopes can be accommodated within a single nanoparticle. Moreover, these nanocarriers can also provide several additional functions, for instance: i) photothermal effect, ii) load chemotherapeutic drug, iii) radiosensitization in case of high-Z nanoparticles, and iv) real-time tumor imaging. Hence, they can be helpful in improving the efficacy or optimizing the therapeutic planning of internal RT or systemic therapy. Radio-NPs, injected intratumorally, can directly deliver radiation dose to the tumor like BT; and this technique is termed as nanobrachytherapy.

In this article, we review the recent progress in the radiosynthesis of the nanoparticles and their use for nanobrachytherapy applications. Recent progress on the i) radiosynthesis methods, ii) selection of a radionuclide for nanobrachytherapy application, iii) modes of internalization of nanocarriers, and iv) the most recent preclinical and dosimetric studies on BT are discussed.

The intratumoral (i.t.) injection of radio-NPs for nanobrachytherapy applications is associated with several challenges and shortcomings. The two main obstacles that have impeded the clinical translation of radio-NPs are i) leakage of fraction of injected radio-NPs from the tumor and ii) inhomogeneous distribution of radioactivity within the tumor post i.t. injection.

Intratumoral retention of radio-NPs is crucial for the therapeutic effectiveness of nanobrachytherapy application and must be maximized. It also reduces the risk of irradiating normal tissues or healthy organs (especially the liver and spleen). Both inhomogeneous intratumoral radioactivity distribution and leakage of radio-NPs from the tumor post injection are caused by irregular tumor vasculature, variable blood and lymph flow, and pressure gradients. Since tumors are unique, the radio-NP leakage ratio and distribution of NPs within the tumor may vary from patient to patient. This will probably make treatment planning and dosimetric computations complicated and challenging. The tumor retention of radio-NPs can be improved by i) functionalizing surface of radio-NPs with tumor-specific biomolecules or ii) co-injecting biocompatible polymers that sequester NPs within the tumor, along with radio-NPs. The delivery systems, injected intratumorally, that can homogeneously distribute radioactivity throughout the tumor volume with minimal leakage have not been developed yet.

Consequently, for clinical translation of intratumorally injected radio-NPs for nanobrachytherapy applications, the injected radio-NPs should i) have high intratumoral retention and ii) homogeneously distribute radioactivity throughout the tumor volume.

Hence, more comprehensive biodistribution studies are required to understand and control the excretion routes of radio-NPs. Furthermore, intratumoral distribution and diffusion of NPs depend on i) tumor architecture and its density; ii) interstitial fluid pressure; iii) tumor vasculature, blood flow, and lymph flow; and iv) specifics of extracellular matrix of the tumor. These factors must be investigated in a wide range of tumors in order to reduce the inhomogeneity in the intratumoral radioactivity distribution. Lastly, for accurate computation of dose distribution at the cellular and subcellular levels within a tumor injected with radio-NPs, the computational model should consider i) *in vivo* microscopic distribution of radio-NPs, ii) complex cell geometry, and iii) distribution of radio-NPs near and within the nucleus. The computational model should also include all possible physics processes that are susceptible to participate in radiation dose distribution at the microscopic scale.

## Author Contributions

BS: initial idea, conceptualization, data collection, and manuscript preparation. LF: manuscript preparation. VT: manuscript preparation. SS: initial idea, basic framework, review of the manuscript, and supervision. TF: supervision and manuscript review. All authors contributed to the article and approved the submitted version.

## Conflict of Interest

The authors declare that the research was conducted in the absence of any commercial or financial relationships that could be construed as a potential conflict of interest.

## Publisher’s Note

All claims expressed in this article are solely those of the authors and do not necessarily represent those of their affiliated organizations, or those of the publisher, the editors and the reviewers. Any product that may be evaluated in this article, or claim that may be made by its manufacturer, is not guaranteed or endorsed by the publisher.
